# Oxidative stress induced in *E*. *coli* by the human antimicrobial peptide LL-37

**DOI:** 10.1371/journal.ppat.1006481

**Published:** 2017-06-30

**Authors:** Heejun Choi, Zhilin Yang, James C. Weisshaar

**Affiliations:** 1 Department of Chemistry, University of Wisconsin-Madison, Madison, WI, United States of America; 2 Molecular Biophysics Program, University of Wisconsin-Madison, Madison, WI, United States of America; Harbor-UCLA Medical Center, UNITED STATES

## Abstract

Antimicrobial peptides (AMPs) are thought to kill bacterial cells by permeabilizing their membranes. However, some antimicrobial peptides inhibit *E*. *coli* growth more efficiently in aerobic than in anaerobic conditions. In the attack of the human cathelicidin LL-37 on *E*. *coli*, real-time, single-cell fluorescence imaging reveals the timing of membrane permeabilization and the onset of oxidative stress. For cells growing aerobically, a CellROX Green assay indicates that LL-37 induces rapid formation of oxidative species after entry into the periplasm, but before permeabilization of the cytoplasmic membrane (CM). A cytoplasmic Amplex Red assay signals a subsequent burst of oxidative species, most likely hydrogen peroxide, shortly after permeabilization of the CM. These signals are much stronger in the presence of oxygen, a functional electron transport chain, and a large proton motive force (PMF). They are much weaker in cells growing anaerobically, by either fermentation or anaerobic respiration. In aerobic growth, the oxidative signals are attenuated in a cytochrome oxidase–*bd* deletion mutant, but not in a –*bo*_3_ deletion mutant, suggesting a specific effect of LL-37 on the electron transport chain. The AMPs melittin and LL-37 induce strong oxidative signals and exhibit O_2_-sensitive MICs, while the AMPs indolicidin and cecropin A do not. These results suggest that AMP activity in different tissues may be tuned according to the local oxygen level. This may be significant for control of opportunistic pathogens while enabling growth of commensal bacteria.

## Author summary

Antimicrobial peptides play a significant role in the innate immune response of plants and animals, including humans. While it is well known that AMPs can permeabilize bacterial cell membranes, a growing body of evidence indicates that they cause a variety of additional deleterious effects. Here we use single-cell imaging methods to study the induction of oxidative stress in live *E*. *coli* by several natural cationic AMPs, including the human cathelicidin LL-37. Strong fluorescence signals indicative of oxidative stress correlate with smaller minimum inhibitory concentrations (MICs) in aerobic vs anaerobic growth conditions. A detailed mechanistic study suggests that LL-37 disrupts the proper flow of electrons through the electron transport chain, releasing oxidative species into the periplasm. Based on these results, we suggest that the degree of aeration in different tissue types may be used by the host to modulate AMP efficacy.

## Introduction

Antimicrobial peptides (AMPs, also called host-defense peptides) play a number of important roles in the innate immune response of plants and animals [1]. Important human AMPs include the cathelidicin LL-37 and the defensins [2]. In humans, AMPs are constitutively expressed in phagocytes, including macrophages, neutrophils, and dendritic cells [3, 4]. When a pathogen attacks the host, phagocytes initially envelope the invading microbes in internal phagosomes [5]. The phagosome fuses with lysosomes to form the phagolysosome. Presence of the pathogen stimulates a “respiratory burst” in the phagocyte, leading to synthesis of harmful reactive oxygen species (ROS) within the phagolysosome [6, 7]. In parallel, AMPs stored in granules are released into the phagolysosome, where their high concentration likely contributes to direct killing of the invading pathogen. AMPs are also released from the phagocyte into surrounding tissue and the bloodstream. In addition to directly attacking pathogens, these external AMPs serve a variety of immunoregulatory functions [8, 9].

Most AMPs are cationic and amphipathic. They are attracted to the anionic outer surfaces of bacterial cells and, at sufficient concentration, permeabilize bacterial membranes. In early studies, the halting of growth of bacterial pathogens by AMPs was typically attributed to permeabilization of the cytoplasmic membrane (CM), with concomitant loss of the proton motive force (pmf), loss of critical small molecules, and halting of ATP production. However, over the past 15 years, many studies have shown that AMPs cause a variety of deleterious biophysical and biochemical effects in bacterial cells, including interference with cell wall biosynthesis, DNA replication, transcription, and translation [10–13]. Induction of reactive oxygen species (ROS) has received little attention as a potentially important aspect of AMP action against bacterial cells [14].

We recently used time-resolved, single-cell fluorescence microscopy [15–19] to show that the hybrid synthetic peptide CM15 (15 aa long, net +6 charge) induces oxidative stress within seconds of contact with *E*. *coli* growing in aerobic conditions [20]. The minimum inhibitory concentration (MIC) was 20-fold higher in anaerobic (fermentation) conditions than in aerobic growth, suggesting that induction of oxidative stress may be a significant growth-halting mechanism. Additional evidence from the oxidation sensitive dye CellROX Green and an intracellular Amplex Red assay suggested that CM15 may interfere with the electron transport chain, possibly leading to formation of superoxide (•O_2_^–^) and hydroxyl radical (•OH) as well as hydrogen peroxide (H_2_O_2_) [21]. We observed analogous effects of oxygen for **MM**_**63**_:**CHx**_**37**_, a potent example of a highly cationic, random β-peptide copolymer (mean chain length 35 units, 63% cationic sidechains) [22]. The MIC of this copolymer against *E*. *coli* is at least 8-fold lower in aerobic than in anaerobic conditions.

Both CM15 and **MM**_**63**_:**CHx**_**37**_ are synthetic peptides. To get a better sense of the generality of these phenomena, here we extend our studies of oxidative effects to four natural AMPs (Table 1): LL-37 (human cathelicidin, α-helical, 37 aa long, net +6), cecropin A (moth, α-helical, 37 aa long, net +7)), melittin (bee, α-helical, 26 aa long, net +6), and indolicidin (bovine, extended structure, 13 aa long, net +4). LL-37 and melittin exhibit significantly lower MICs against *E*. *coli* in aerobic vs anaerobic (fermentation) conditions, and they both induce strong fluorescence signals indicative of oxidative stress. In contrast, for cecropin A and indolicidin the MIC is the same in aerobic and anaerobic conditions. Correspondingly, they induce much weaker fluorescence signals.

**Table 1:** Antimicrobial peptides and their MICs for *E*. *coli* MG1655 in aerobic growth vs anaerobic fermentation in EZRDM at 30°C.

Antimicrobial Peptide	Sequence	Net Charge	MIC (μM) ^1^	
			Aerobic	Fermentation
**LL-37**	**LLGDFFRKSKEKIGKEFKRIVQRIKDFLRNLVPRTES**	+6	4	16
**Cecropin A**	**KWKLFKKIEKVG** **QNIRDGIIKAGPAV** **AVVGQATQIAK-NH**_**2**_	+7	0.9	0.9
**Melittin**	**GIGAVLKVLTTGL-PALISWIKRKRQQ-NH**_**2**_	+6	5	40
**CM15**	**KWKLFKKIGAVLKVL-NH**_**2**_	+6	5	100
**Indolicidin**	**ILPWKWPWWPWRR-NH**_**2**_	+4	32	32

^1^ MIC values were reproducible to the same factor of two in the dilution series for multiple repeats of the assay.

In addition, we provide a detailed, single-cell comparison of LL-37 attacking *E*. *coli* growing under conditions of aerobic respiration, anaerobic fermentation, and anaerobic respiration. In aerobic growth, a burst of oxidative species is induced already on access of LL-37 to the periplasm, *i*.*e*., well before the cytoplasmic membrane is permeabilized to the dye Sytox Orange. The mechanism may involve interference with proteins of the electron transport chain (ETC), leading to improper release of superoxide (•O_2_^–^) into the periplasmic space. Mutation studies suggest that LL-37 targets the cytochrome oxidase-*bd* complex, but not the cytochrome oxidase-*bo*_3_ complex. A subsequent burst of oxidative species, detected by an intracellular Amplex Red assay sensitive to H_2_O_2_, rose sharply at the moment of CM permeabilization. For cells growing by anaerobic fermentation or by anaerobic respiration using NO_3_^–^ as terminal electron acceptor, no such signals of abrupt oxidative events were observed. However, the CellROX Green and Amplex Red assays are insensitive to oxidative nitrogen-containing radicals, so that oxidative damage might still be occurring.

These new results suggest the possibility that the host may use the degree of tissue aeration for selective control of the potency of AMPs. The present work indicates that LL-37 is most potent against *E*. *coli* in oxygen-rich conditions. Earlier work found the same effect for the human beta defensin hBD-3, but the opposite effects for hBD-1 [23]. Reduction of the Cys-Cys linkages in hBD-1, which converts the globular oxidized structure to a linear structure, greatly enhanced its antimicrobial activity against anaerobic Gram positive species. Tuning of cationic AMP activity according to local redox conditions may prove to be important in controlling opportunistic pathogens while enabling growth of commensal bacteria.

## Results

### Minimum inhibitory concentration of some AMPs depend on growth conditions

We have measured MICs in aerobic and anaerobic fermentation conditions using a series of two-fold dilutions for the four natural AMPs in the same rich, chemically defined EZRDM medium at 30°C (Table 1). The MIC in anaerobic fermentation conditions is 4-fold higher for LL-37 and 8-fold higher for melittin. Multiple experimental runs produce the same MIC value within the resolution of the two-fold dilution steps, indicating that 4-fold and 8-fold differences are significant. For cecropin A and for indolicidin, the MIC is the same in aerobic and fermentation conditions. Evidently the activity against *E*. *coli* of some, but not all, natural AMPs is enhanced by the presence of oxygen. For LL-37, the primary focus of this study, we also measured the MIC in conditions enabling anaerobic respiration (no oxygen, but supplemented with 10 mM KNO_3_; Table 2). The MIC is 12 μM, three times higher than in aerobic respiration.

**Table 2:** Bacterial strains, with doubling times and MIC values for LL-37.^1^.

Strain^2^	Description	Growth Condition	Doubling Time (min) ^1^	MIC (μM) ^1^
WT	MG1655	Aerobic	50 ± 3 ^3^^,^^4^	4
JCW10	exports GFP to periplasm	Aerobic	51 ± 3 ^3^	–
ZY01	expresses APEX2 from plasmid in MG1655	Aerobic	53 ± 3 ^3^	–
Δ*cyoABCDE*	cytochrome oxidase-*bo*_3_ deletion mutant of MG1655	Aerobic	53 ± 3 ^3^	4
Δ*cydAB*	cytochrome oxidase-*bd-I* deletion mutant of MG1655	Aerobic	45 ± 3 ^3^	4
ZY02	expresses APEX2 in Δ*cyoABCDE* strain	Aerobic	–	–
WT	MG1655	Anaerobic	53 ± 3 ^4^	16
WT	MG1655	Anaerobic + 10 mM KNO_3_	45 ± 3 ^4^	12

^1^ Doubling times and six-hour MIC values for LL-37 in EZRDM at 30°C. MIC values were reproducible to the same factor of two in the dilution series for multiple repeats of the assay. Doubling time precision is ±3 min, as judged from repeat experiments.

^2^ See Methods for description of how strains were made.

^3^ Determined in bulk cultures by OD measurements; see Methods.

^4^ Determined by measuring cell length vs time using phase-contrast microscopy; see [18].

The doubling times for MG1655 growing in EZRDM at 30°C under aerobic respiration, anaerobic respiration with KNO_3_, and fermentation conditions are similar, all in the range 45–53 min (Table 2). Earlier work in M9 medium supplemented with hydrolyzed casein [24] found that *E*. *coli* maintained a substantial proton motive force (pmf) in all three growth conditions (–160 mV for aerobic respiration, –144 mV for anaerobic respiration using NO_3_^–^, and –117 mV for fermentation). These pmf values may not be transferable to our strain and growth conditions.

### Bactericidal effects of LL-37 at the minimum inhibitory concentration in aerobic growth conditions

We chose the human cathelicidin LL-37 for a detailed time-dependent, single-cell study of antimicrobial action. First we investigated the extent to which LL-37 in aerobic conditions at the 6-hour MIC of 4 μM causes cell death (irreversible halting of growth) on the timescale of our microscopy measurements, typically 30–60 min. We monitored cell killing activity using a conventional cell survival assay. A mid-log phase culture of MG1655 *E*. *coli* was incubated with LL-37 at 4 μM, sampled after 30 min, 1 hr, and 2 hr of incubation, and serially diluted over the range 5 x 10^6^ to 5 cells/mL. One μL of the diluted sample was spot-plated onto a 3% LB-agar plate for overnight growth at 30°C. In control experiments, no LL-37 was added prior to plating and incubation. At 4 μM LL-37, we observe a significant decrease in colony formation after 30 min of incubation when compared to the control (S1 Fig). After 1-hr incubation at 4 μΜ, essentially no colonies formed, even for the least diluted sample. These results were reproducible over three trials. According to classic clinical microbiological definitions, this indicates that the MIC and the minimum bactericidal concentration (MBC) for LL-37 are essentially the same. At 4 μM of LL-37, both growth inhibition and cell death occur within a 1-hr period. This indicates that the single-cell signs of LL-37 attack as observed under the microscope on the 30–60 minute timescale are likely relevant to cell killing activity.

### Sequence of membrane permeabilization events in aerobic growth conditions

For individual *E*. *coli* cells, time-lapse microscopy can determine the timing of the slowing or halting of cell growth, of outer membrane permeabilization, and of cytoplasmic membrane permeabilization following the onset of flow of LL-37 (Methods). Warm (30°C), aeriated EZRDM growth medium flows continuously across the plated cells. Over an observation period of 30–60 min, we alternate phase contrast images with fluorescence images (either one or two colors) and make time-dependent, quantitative measurements of cell length and total fluorescence intensity. In the first set of measurements, the *E*. *coli* cells express GFP that is exported to the periplasm by the twin-arginine transport (Tat) system [25]. This produces a characteristic halo image [26]. The medium contains 5 nM of the DNA stain Sytox Green, which becomes fluorescent on crossing both membranes and binding to the chromosomal DNA within the cytoplasm. We directly observe cell length vs time (from phase contrast), the onset of permeabilization of the OM to periplasmic GFP (observed as loss of the green halo surrounding the cytoplasm), and the onset of permeabilization of the CM to Sytox Green (from green staining of the nucleoids).

**Fig 1:**
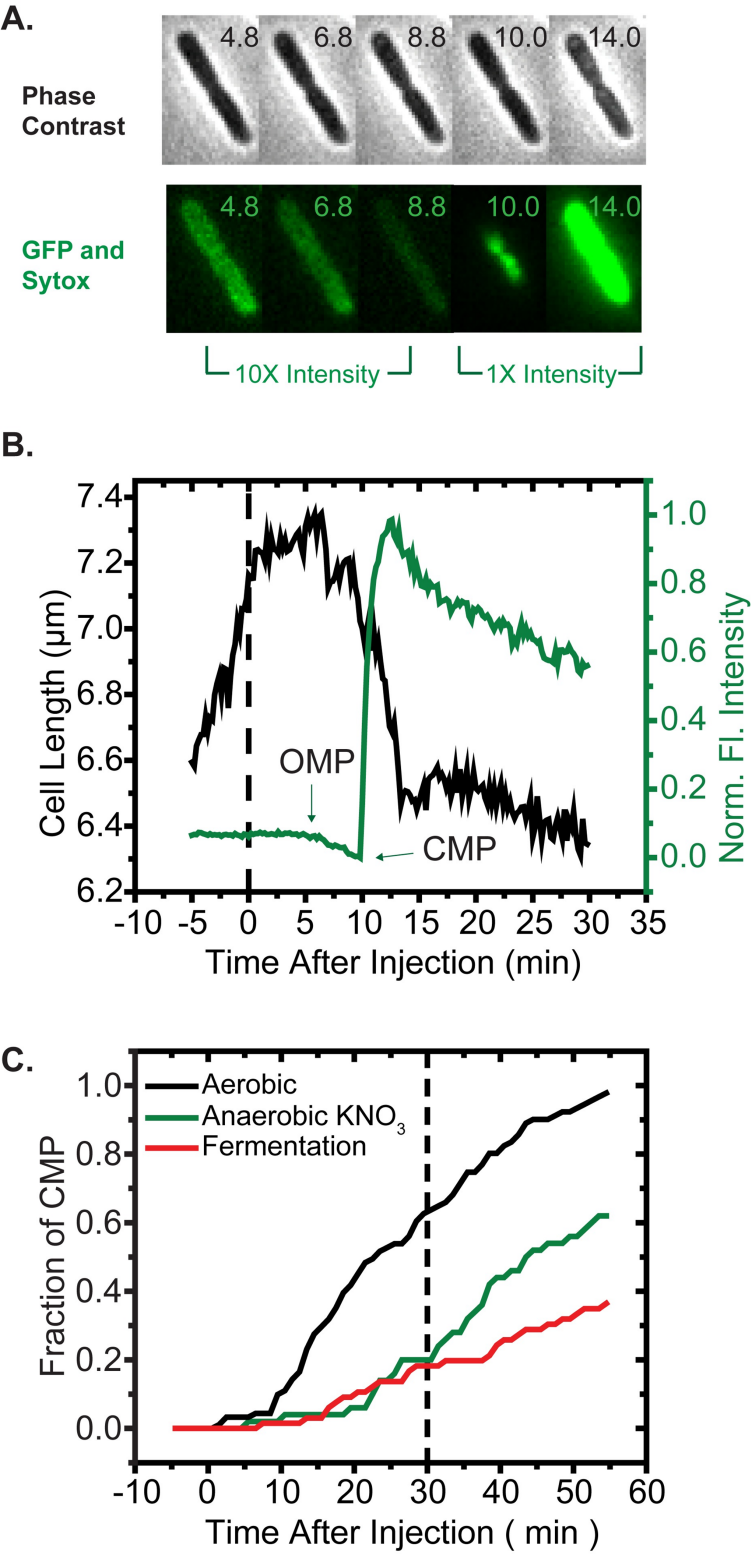
**(**A) Phase contrast and green fluorescence snapshots of single *E*. *coli* cell expressing periplasmic GFP in aerobic growth conditions, following addition of 4 μM LL-37 (the 6-hr aerobic MIC) with 5 nM Sytox Green. Times in minutes after injection of LL-37 at *t* = 0. (B) Time dependence of cell length (from phase contrast images) and total GFP and Sytox green fluorescence intensity for the cell shown in (A). Time of outer membrane permeabilization (OMP) and cytoplasmic membrane permeabilization (CMP) as shown. The initial decrease of fluorescence is due to loss of GFP from the periplasm to the surroundings. The subsequent burst of fluorescence is due to access of Sytox green to the cytoplasm upon CM permeabilization. (C) Cumulative distribution function (CDF) of the fraction of cells that have undergone CM permeabilization (onset of Sytox green fluorescence) vs time after the injection of 4 μM LL-37. Comparison for growth in aerobic respiration, anaerobic respiration using NO_3_^–^, and fermentation.

At *t* = 0, we initiate flow of 4 μM LL-37 (the 6-hr MIC) in aerated medium through the microfluidics observation chamber. For the example cell in Fig 1, the growth rate (slope of the plot of cell length vs time) begins to decrease immediately after injection of LL-37. For at least 90% of the cells in a typical field of 50 cells, we observe gradual slowing or abrupt halting of growth within 10 min of injection. Over the first 30 min after injection of LL-37, 60% of the cells lose the “halo” of periplasmic GFP, indicating OM permeabilization to GFP (Fig 1C). During the same 30 min, the other 40% of the cells exhibit an attenuated growth rate, yet continue to elongate without loss of periplasmic GFP. Loss of GFP begins over a wide range of times (2–50 min). Once it begins, complete loss of GFP occurs fairly quickly, over the subsequent 2–3 min. The onset of GFP intensity loss almost always coincides with moderate shrinkage of cell length. As observed before [19], obviously septating cells tend to undergo OM permeabilization earlier than apparently non-septating cells.

For the 60% of cells that undergo OM permeabilization before *t* = 30 min, growth halts. A new signal from Sytox Green begins to rise within 5 min of the OM permeabilization event (example in Fig 1 and S1 Movie). Green fluorescence evolves in the cytoplasm in a spatial pattern reminiscent of the distribution of the *E*. *coli* nucleoids, indicating CM permeabilization to Sytox Green. Permeabilization of the CM correlates in time with additional shrinkage of cell length, presumably due to loss of osmolytes from the cytoplasm. The 40% of cells that continued to elongate slowly for the first 30 min did not display a Sytox Green signal during that period, indicating that both the CM and the OM remained intact. However, eventually almost all cells exhibit both OM and CM permeabilization within a 1-hr period after LL-37 addition, as shown by the cumulative distribution function of the lag times to CM permeabilization (Fig 1C). The timescale of the halting of growth observed in these single-cell permeabilization experiments at the MIC is consistent with the results of the bulk, time-lapse bactericidal assay.

### Overview of real-time oxidative stress signals

The MIC data suggest that the halting of growth at 4 μM of LL-37 is mediated by oxygen. Our working hypothesis is that LL-37 induces formation of harmful reactive oxygen species (ROS). In an earlier study of CM15 [20], we developed two single-cell, real-time fluorescence measurements that monitor oxidative stress using the dyes CellROX Green and Amplex Red. CellROX Green (Life Technologies) is a proprietary, permeable, non-fluorescent, oxidation-sensitive dye. Oxidation produces a species we call CellROX*, which fluoresces in the green, but only when bound to ds-DNA. *In vitro*, CellROX Green is sensitive to superoxide (•O_2_^–^) and to hydroxyl radical (•OH), but not to hydrogen peroxide (H_2_O_2_) or to a variety of other oxidants including peroxynitrite (ONOO^–^), NO, and hypochlorite (OCl^–^). In the cellular environment, other species such as high-valence Fe centers could also oxidize CellROX Green [27, 28]. Amplex Red is a permeable dye whose reaction with H_2_O_2_ is catalyzed by the non-native peroxidase APEX2, expressed in the cytoplasm from a plasmid [29, 30]. The product is resorufin, which fluoresces in the red. The specificity of the enzymatic reaction strengthens the assumption that resorufin fluorescence signals H_2_O_2_ formation.

We monitor oxidative stress by measuring single-cell fluorescence of CellROX* or resorufin (in cells expressing APEX2) as a function of time after LL-37 addition. The duration of each complete imaging cycle is 12 s for one-color imaging and 6 s for two-color imaging.

### Onset of CellROX* fluorescence occurs on entry of LL-37 into the periplasm

**Fig 2:**
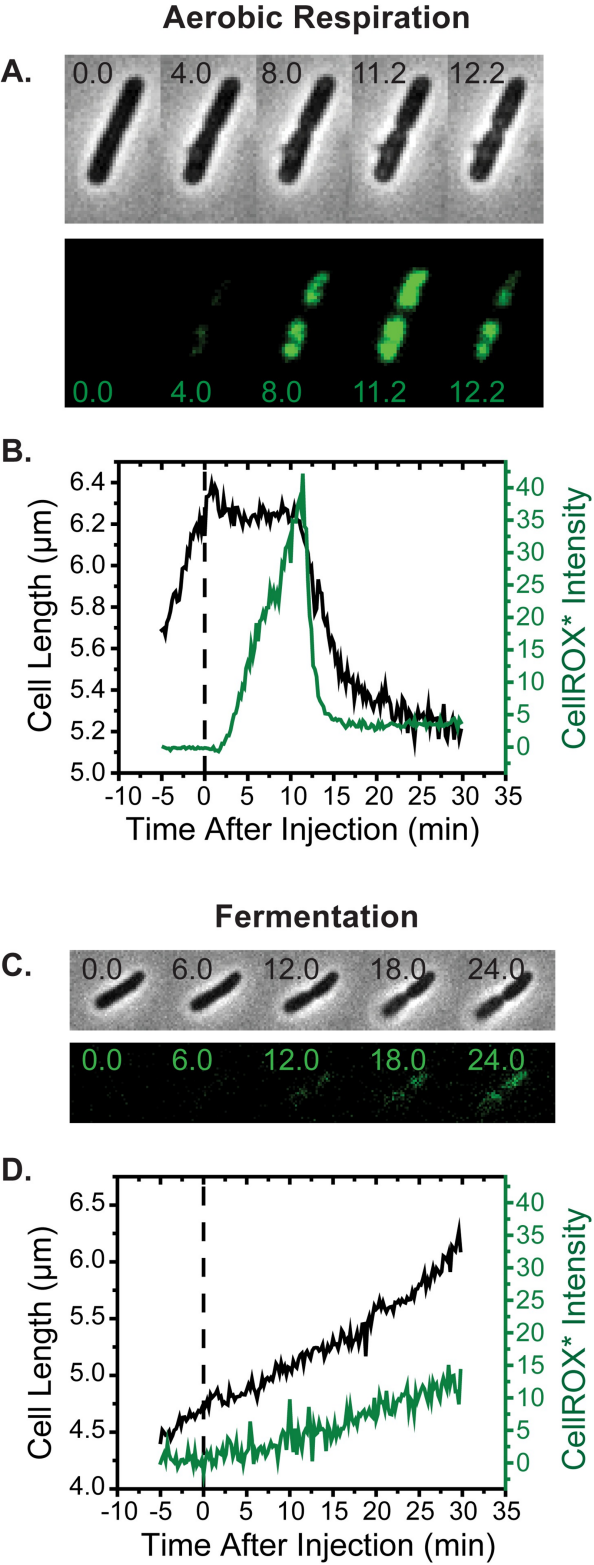
(A) Phase contrast and green fluorescence snapshots vs time after initiating flow at *t* = 0 of 4 μM LL-37 and 2.5 μM CellROX Green over MG1655 *E*. *coli* growing in aerobic conditions. Periplasmic GFP is not present. (B) Cell length and total CellROX* fluorescence intensity vs time for the cell shown in (A). (C and D) Same as panels (A and B), but cells are growing in anaerobic conditions under fermentation.

First we carried out the CellROX* assay in aerobic conditions using 4 μM of LL-37 (the aerobic MIC). At *t* = 0 the flow was switched to medium including LL-37 and 2.5 μM CellROX Green. Laser intensities and imaging conditions were held constant, enabling quantitative intensity comparisons across different experiments. More than 90% of 171 cells from three repeats of the experiment exhibit attenuation of growth rate or abrupt shrinkage within 10 min of injection of LL-37 (example in Fig 2A and 2B and S2 Movie), as was observed in the periplasmic GFP experiments. For the particular cell in Fig 2, halting of growth and mild shrinkage occurs shortly after *t* = 0. At *t* = 2 min, CellROX* fluorescence intensity begins to increase gradually; the intensity continues to rise for about ten minutes, when it turns sharply downward and decays to a non-zero plateau. At the same moment, cell length begins a second period of gradual shrinkage. For all cells whose length begins to decrease at *t* < 30 min, we eventually observe a sudden decrease in CellROX* signal to a non-zero plateau. We show below that this decrease typically correlates in time with the moment of CM permeabilization to the DNA stain Sytox Orange. Those cells that continue to grow slowly over the first 30 min (no CM permeabilization), exhibit a much weaker CellROX* signal that increases slowly throughout the observation period.

**Fig 3:**
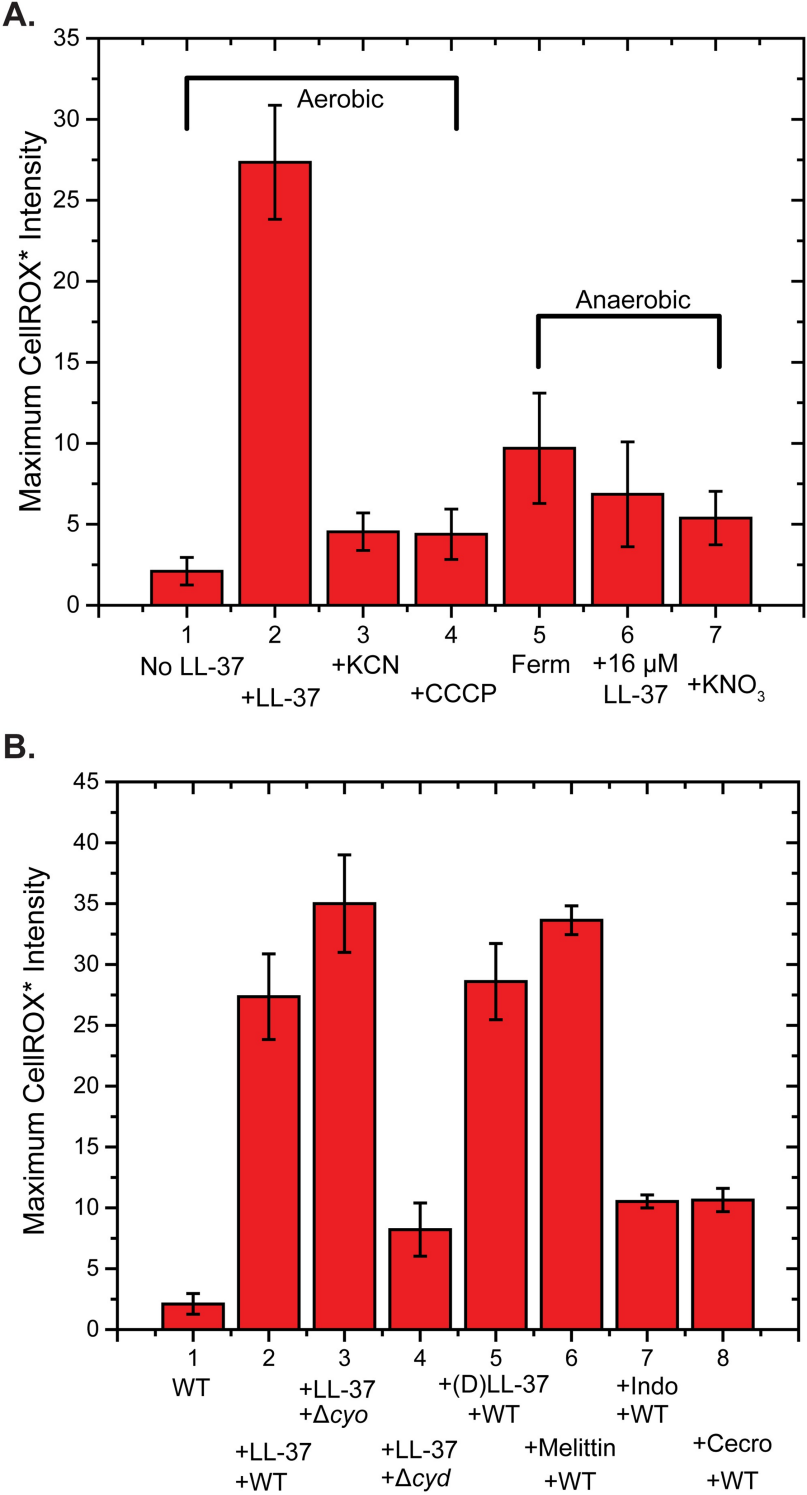
Comparison of average maximum CellROX* intensity over 30 min under different conditions. **(A)** (1) Normal aerobic growth (23 cells); no LL-37. (2) Addition of 4 μM LL-37 in aerobic conditions (58 cells). (3) Pre-treatment with KCN, followed by 4 μM LL-37 in aerobic conditions (62 cells). (4) Pre-treatment with CCCP, followed by 4 μM LL-37 in aerobic conditions (90 cells). (5) Addition of 4 μM LL-37 to cells growing in anaerobic, fermentation conditions (25 cells). (6) Addition of 16 μM LL-37 to cells growing in anaerobic, fermentation conditions (34 cells). (7) Addition of 4 μM LL-37 to cells growing in anaerobic conditions with respiration using NO_3_^–^ (23 cells). In cases (2) and (6), the maximum intensity is that at the peak of the gradually rising and sharply falling signal, as in Fig 2B. In all other cases, the maximum is that of a slowly rising signal at *t* = 30 min, as in Fig 2D. **(B)** Comparison of average maximum CellROX* intensity over 30 min following addition of AMP as indicated. (1) Normal aerobic growth of WT MG1655 cells; no LL-37 (23 cells). (2) Addition of 4 μM LL-37 to WT MG1655 cells in aerobic growth (58 cells). (3) Addition of 4 μM LL-37 to Δ*cyoABCDE* MG1655 cells (cytochrome oxidase-*bo*_3_ deletion strain) in aerobic growth (50 cells). (4) Addition of 4 μM LL-37 to Δ*cydAB* MG1655 cells (cytochrome oxidase-*bd* deletion strain) in aerobic growth (29 cells). (5) Addition of 4 μM of the enantiomer (*D*)LL-37 to WT MG1655 cells in aerobic growth. (30 cells) (6) Addition of 10 μM melittin to WT cells in aerobic growth (42 cells). (7) Addition of 32 μM indolicidin to WT cells in aerobic growth (42 cells). (8) Addition of 0.9 μM cecropin A to WT cells in aerobic growth (49 cells).

Control experiments indicate that the peak CellROX* signal induced by LL-37 from 58 analyzed cells is on average ten times larger than the magnitude of the slowly rising green fluorescence signal observed at *t* = 30 min in the absence of LL-37 (Fig 3A). These data suggest that the strong CellROX* fluorescence begins to rise when LL-37 penetrated the OM and gains access to the periplasm. Experiments using two-color imaging of CellROX* and Rhodamine B-LL-37 will corroborate this inference.

**Fig 4:**
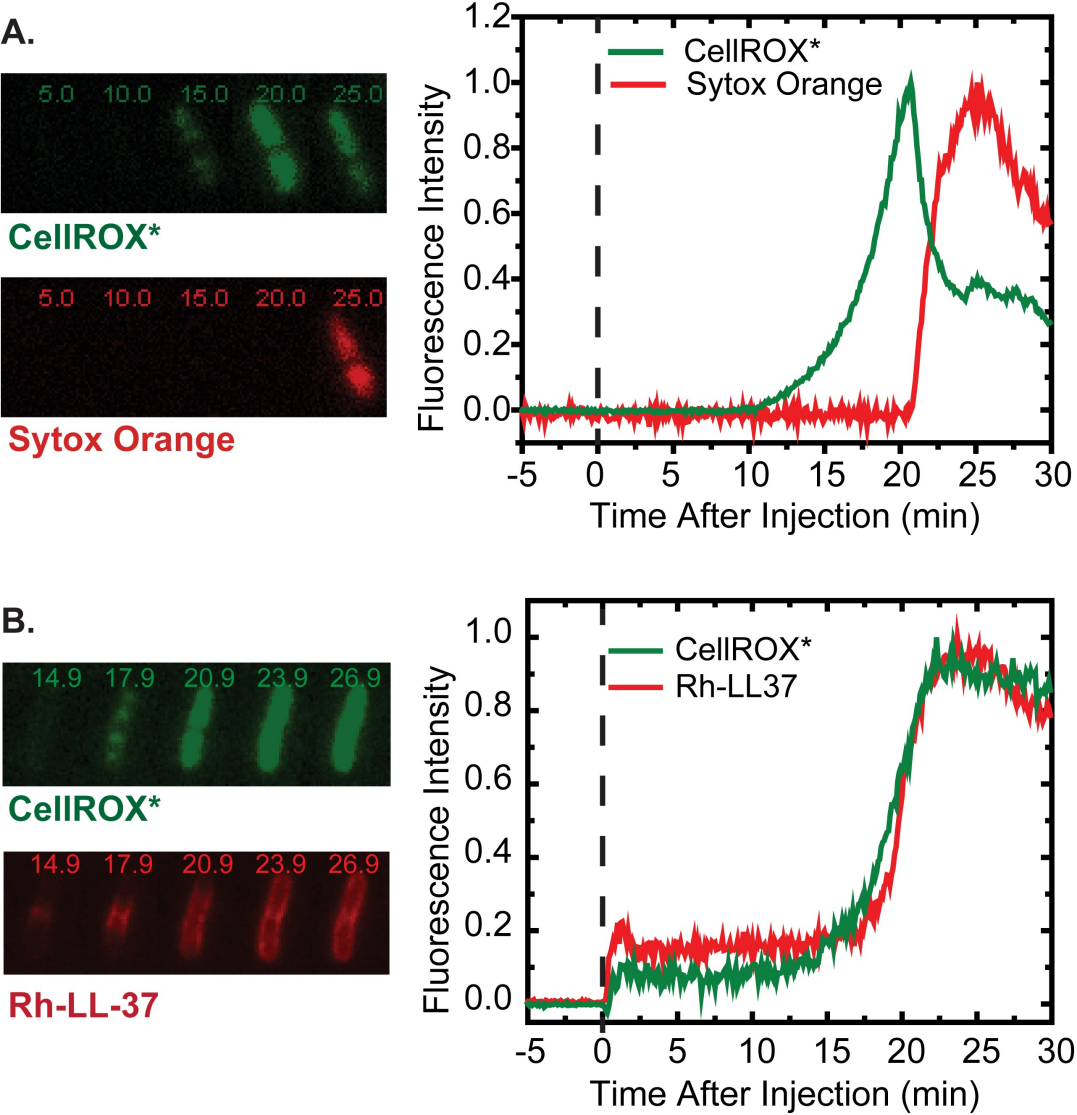
(A) Relative timing of CellROX* and Sytox Orange signals from two-color imaging. Aerobic growth conditions. *Left*: Snapshots of green and red fluorescence images of a single MG1655 cell after treatment with 4 μM LL37, 2.5 μM CellROX Green, and 5 nM Sytox Orange. Times in minutes after injection. Aerobic growth conditions. *Right*: CellROX* and Sytox Orange total fluorescence intensity vs time for cell shown at left. (B) Relative timing of CellROX* and Rh-LL-37 signals from two-color imaging. *Left*: Snapshots of green and red fluorescence images of a single MG1655 cell after treatment with 8 μM Rh-LL-37 plus 25 μM CellROX Green at *t* = 0. Times in minutes after injection. Aerobic conditions. *Right*: CellROX* and Rh-LL-37 total fluorescence intensity vs time for the cell shown at left.

To directly confirm that CellROX* begins to rise before CM permeabilization, we carried out two-color fluorescence experiments in aerobic conditions, injecting both 2.5 μM CellROX Green and 5 nM of the DNA stain Sytox Orange into the growth medium at *t* = 0. Observations were then carried out for 30 min. A typical example of the two fluorescence traces from a single cell that undergoes CM permeabilization during the observation period is shown in Fig 4A. See also S3 Movie. A strong CellROX* fluorescence begins to rise some 10 min before the abrupt onset of Sytox Orange fluorescence, which in turn marks the moment when the CM is permeabilized. The CellROX* signal then typically decreases abruptly by about 60% and stabilizes at a lower value. As before, the abrupt decrease in CellROX* fluorescence occurred in all cells exhibiting CM permeabilization over 30 min. Most often the CellROX* signal decrease begins within 1 min of CM permeabilization, but in about 1/3 of 39 cells the decrease begins 2–15 min after CM permeabilization. A histogram in shown in S6 Fig.

Additional evidence that the onset of strong CellROX* fluorescence coincides with entry of LL-37 into the periplasm comes from two-color imaging experiments using CellROX Green and a red fluorescent variant of the peptide, Rh-LL-37. Earlier we showed that Rh-LL-37 and unlabeled LL-37 have the same MIC vs MG1655 *E*. *coli*, although membrane permeabilization events occurred somewhat more slowly for Rh-LL-37 [19]. Under aerobic growth conditions, at *t* = 0 we flowed 25 μM CellROX Green plus 8 μM Rh-LL-37 across plated *E*. *coli* cells. A representative result is shown in Fig 4B. As before [19], weak red fluorescence from Rh-LL-37 initially coats all cells uniformly (plateau of red fluorescence at *t* = 2–17 min). We attribute this to binding of Rh-LL-37 oligomers to the lipopolysaccharide (LPS) layer. The weak plateau of green fluorescence is a background signal that also coats all cells uniformly. This background is not CellROX* fluorescence, because it occurs on addition of Rh-LL-37 in the absence of CellROX Green.

Like LL-37, Rh-LL-37 preferentially attacks septating cells. As observed earlier [19], septating cells gradually exhibit a brighter band of red fluorescence that begins at the septal region and slowly spreads to the entire periplasm over 5–10 min (Fig 4B). In salty solution Rh-LL-37 fluorescence is self-quenched due to bundling of multiple helices. We believe that this self-quenching persists during binding to the LPS layer of *E*. *coli*, rendering the initial wave of red fluorescence weak. Entry into the periplasm at the septal region unbundles the helices, causing dequenching of fluorescence and gradual development of the brighter red band. Evidently Rh-LL-37 enters at the septal region and binds strongly to some immobile element of the periplasm, possibly the anionic cross-links within the peptidoglycan layer. As local binding sites become occupied, unbundled Rh-LL-37 slowly migrates towards the tips of the periplasm, observed as a gradual outward spreading of the brighter red band. In earlier work, we showed that Rh-LL-37 binds to purified peptidoglycan [19].

As shown in the example of Fig 4B, the green fluorescence from CellROX* and the brighter band of red fluorescence from Rh-LL-37 in the periplasm rise on the same time scale. This occurred within 30 min for 59 cells from three repeat experiments. We infer that oxidative species are formed gradually, as more and more monomeric Rh-LL-37 copies gain access to the periplasm.

In an important control experiment, in earlier work [20] we found that permeabilization of both the OM and the CM using Triton-X (without addition of AMP) did not enhance CellROX* fluorescence. This shows that CM permeabilization alone is not sufficient to trigger the signals of oxidative stress observed after LL-37 treatment.

### No effect of the enantiomer *D*-LL-37 on CellROX* signal level

To test for the importance of LL-37 stereochemistry on the magnitude of oxidative effects, we repeated the CellROX* assay in aerobic conditions using 4 μM of the all-*D* stereoisomer of LL-37. The average peak CellROX* signal level was the same within experimental error (Fig 3B). In addition, our earlier work found the same MIC for *D-* and *L*-LL-37 [19].

### Attenuation of CellROX* response by pre-treatment with cyanide

Our working hypothesis is that in aerobic conditions, LL-37 causes formation of ROS (most likely •O_2_^–^) in the periplasm by disrupting the electron transport chain. The disruption begins when LL-37 gains access to the periplasm, which also affords access to the outer leaflet of the CM. The electron transport chain employs a series of membrane proteins embedded in the CM [31]. For exponential growth in aerobic conditions, the primary pathway runs through the two NADH dehydrogenases NDH-I and NDH-II (with complex II dominant), passes through ubiquinone (UB), and terminates at the cytochrome oxidase-*bo*_3_ complex [32]. Depending on the level of oxygenation, some fraction of the electron flux terminates at the alternative cytochrome oxidase-*bd* complex. The–*bd* complex has much higher affinity for O_2_ than does–*bo*_3_; its expression level increases with decreasing O_2_ concentration in the growth medium. The terminal oxidase converts O_2_ to H_2_O, transferring protons to the periplasm and helping to maintain the proton motive force. At sufficient concentration, CN^−^binds to the key heme iron in both the -*bo*_3_ and the–*bd* complex, blocking O_2_ binding, halting oxidative respiration and cell growth, and greatly diminishing the proton-motive force [21].

To test whether aerobic respiration is a prerequisite for LL-37-induced generation of the oxidative stress signals, we pre-incubated WT MG1655 *E*. *coli* cells with 1 mM KCN for 5 min prior to injection of 4 μM LL-37 in aerated growth medium. According to previous studies of reconstituted respiration *in vitro*, at this concentration the–*bo*_3_ complex is strongly inhibited, but the–*bd* complex is more weakly inhibited [33]. The inhibiting concentration *in vivo* is not known.

After pre-treatment for 5 min with cyanide, cells do not grow over a 50-min observation time, suggesting that respiration has been blocked. In two-color imaging experiments, we initiated flow of 4 μM LL-37 with 2.5 μM CellROX Green and 5 nM Sytox Orange at *t* = 0. The flow also contained 1 mM KCN to block respiration continuously. For the typical cell shown in S2 Fig, no significant rise of CellROX* or Sytox Orange fluorescence was observed on a 50-min timescale. Evidently LL-37 induced neither ROS formation nor CM permeabilization. We measured the maximum CellROX* intensity from 62 cells in three separate experiments with and without pre-incubation with KCN (Fig 3A). The KCN pre-treatment attenuates the mean CellROX* fluorescence per cell by at least a factor of 5.

The KCN treatment also greatly reduces the fraction of cells that exhibit significant Sytox Orange fluorescence over 60 min of LL-37 treatment, from ~100% for normally growing cells to ~30% for KCN-treated cells (S2 Fig). This suggests the possibility that formation of oxidative species, possibly ROS, within the periplasm may enhance the ability of LL-37 to permeabilize the CM. Alternatively, reduction of the transmembrane potential may inhibit the ability of LL-37 to permeabilize the CM, as discussed below.

The same pre-treatment of cells with KCN also reduces the cell-killing effects of LL-37. We repeated the LL-37 bactericidal assay after KCN treatment. As shown in S1 Fig, more pre-treated cells survive after 4 μM LL-37 incubation for 30 min, and also for 1 hr. Evidently KCN pre-treatment provides some protection against the deleterious effects of LL-37. However, when 8 μM LL-37 was applied to cells pre-treated with KCN, no growth was observed even after 1 hr.

In a similar vein, pre-treatment of the cells with the protonophore CCCP at 200 μM for 10 min completely halted growth. Subsequent injection of 4 μM of LL-37 and CellROX Green caused only a small, slowly rising CellROX* signal, again five times smaller than the peak signal from cells growing aerobically (Fig 3A).

### Onset of resorufin fluorescence in aerobic conditions follows CM permeabilization

**Fig 5:**
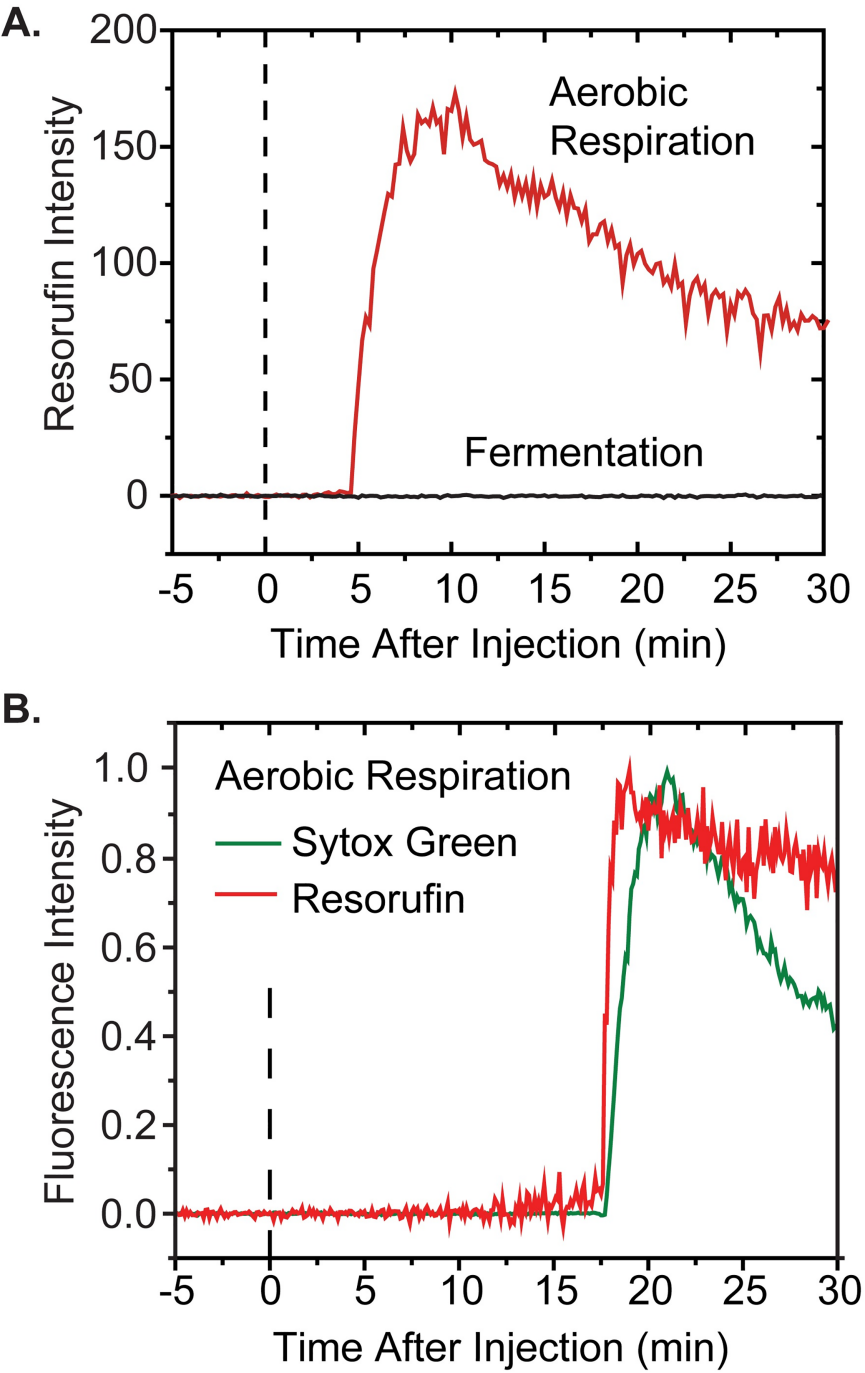
(A) Resorufin total fluorescence intensity vs time in aerobic growth and fermentation conditions. Flow of 4 μM LL-37 and 10 μM Amplex Red over MG1655 *E*. *coli* expressing the peroxidase APEX2 begins at *t* = 0. (B) Aerobic growth conditions. Relative timing of resorufin and Sytox Green signals from two-color imaging. Flow of 4 μM LL-37, 10 μM Amplex Red, and 5 nM Sytox Green over MG1655 *E*. *coli* expressing the peroxidase APEX2 begins at *t* = 0.

We tested for H_2_O_2_ induction in aerobic conditions by repeating the flow experiment using the MG1655 strain expressing the non-native peroxidase APEX2 from a plasmid [30]. At *t* = 0, we flowed 4 μM of LL-37 plus 10 μM Amplex Red and alternated phase contrast and red fluorescence images. Inside some 20% of 121 cells from three repeat experiments, a substantial burst of intracellular resorufin fluorescence is observed (example in Fig 5A and S4 Movie). However, for most cells we observe only a weak intracellular resorufin signal that is difficult to measure above a steadily increasing background of red fluorescence outside the cells (S3 Fig). This strongly suggests that for most cells, resorufin formed in the cytoplasm efficiently escapes the cell envelope. As a result, the intracellular resorufin signal is usually very small.

To determine the exact timing of the onset of the strong, intracellular resorufin signals relative to CM permeabilization, we carried out two-color imaging experiments using Amplex Red and the green fluorescent DNA stain Sytox Green. In the minority of cells that exhibit appreciable intracellular resorufin fluorescence, the abrupt onset of red and green fluorescence is essentially simultaneous (Fig 5B and S5 Movie). Evidently the burst of resorufin is produced promptly after the CM is permeabilized. However, once again the majority of cells show little or no intracellular resorufin signal. And once again a background of red fluorescence in the surround rises gradually over time, suggesting that while most or perhaps all cells are producing H_2_O_2_ in the cytoplasm, the resulting resorufin usually escapes the cell envelope efficiently (S3 Fig). This makes sense; the OM is typically permeabilized to GFP and smaller species long before the CM is permeabilized to Sytox and other small molecules.

To summarize, the typical behavior in time of the CellROX* and resorufin fluorescence signal in aerobic conditions is very different. In most cells, CellROX* fluorescence rises gradually as LL-37 slowly gains entry to the periplasm (before CM permeabilization), and then decreases abruptly by a factor of two or more at the moment of CM permeabilization (Fig 2). In contrast, intracellular resorufin fluorescence rises abruptly at the moment of CM permeabilization (Fig 5). This is observed only in a minority of cells, but red background fluorescence rises gradually over the 30-min experiment.

### Smaller signals of oxidative stress on cytochrome oxidase–*bd* deletion mutant strain

To test the possibility that LL-37 is causing release of oxidants by perturbing proper function of cytochrome oxidase-*bo*_3_ or of cytochrome oxidase-*bd*, we carried out a limited number of microscopy experiments on the deletion mutant strains Δ*cyoABCDE* (–*bo*_3_ deletion mutant) and Δ*cydAB* (–*bd* deletion mutant). The strains exhibit aerobic doubling times of 53 min and 45 min, respectively, similar to WT cells (Table 2). The 6-hr MICs are both 4 μΜ (Table 2), the same as the WT strain. The expression levels of the–*bo*_3_ and–*bd* oxidases vary with the availability of O_2_ in the medium. The–*bo*_3_ oxidase, which binds O_2_ more weakly, is more abundant when the level of dissolved O_2_ is high [31]. The–*bd* oxidase, which binds O_2_ more strongly, is more abundant for low O_2_ concentrations.

As shown for the typical cell in S4 Fig, in aerobic growth the Δ*cyoABCDE* mutant exhibited strong signals in the CellROX* after injection of LL-37 at 4 μM. The mean CellROX* signal level is slightly larger than for the WT strain (Fig 3B). The same mutant also showed strong resorufin signals in the Amplex Red assay, comparable to the signal shown in Fig 5A (S7 Fig). However, the CellROX* signal level for the Δ*cydAB* mutant strain is only 27% that of the WT strain (Fig 3B). This result implicates cytochrome oxidase-*bd* in the mechanism by which LL-37 induces oxidative stress on accessing the periplasmic space.

### Smaller signals of oxidative stress in growth under anaerobic fermentation conditions

No known terminal electron acceptors are present in the standard EZRDM medium. Cells growing in EZRDM with glucose as carbon source but without oxygen (Methods) and without added nitrate carry out fermentation, synthesizing ATP by glycolysis. The doubling time is essentially the same as in aerobic conditions (Table 2), and the pmf is likely reduced by about 25% [32]. At *t* = 0, we initiated flow of 2.5 μM CellROX Green and 4 μM unlabeled LL-37 (the aerobic MIC) over wild-type *E*. *coli* growing in fermentation conditions. The rate of cell growth, as judged by cell length in phase contrast images, decreased early on, much as it did in aerobic conditions. However, on the 30-min timescale, under anaerobic conditions 80% of 134 cells from three separate experiments continued to grow, albeit more slowly (example in Fig 2C and 2D). In comparison, on the same 30-min timescale under aerobic conditions only 40% of the cells continued to grow.

In these fermentation experiments with LL-37, a small green fluorescence signal, possibly due to CellROX*, was typically observed to rise slowly over 30 min as most cells continued to grow (Fig 2C and 2D). Averaged over 20 cells, the maximum green fluorescence intensity achieved during the 30-min observation period was three times smaller in fermentation than in aerobic growth conditions (Fig 3A). There was no abrupt increase in green fluorescence. In contrast, the green signal in aerobic conditions rises more rapidly (over 5–10 min vs 30 min, Fig 2B vs 2D).

We repeated the one-color imaging experiments in fermentation conditions using 4 μM LL-37 along with either Sytox Green or CellROX Green. Over the first 30 min 20% of the 92 cells from three separate experiments exhibited cytoplasmic membrane permeabilization to Sytox Green (Fig 1C) and a halting of growth. Importantly, in the CellROX Green experiments no abrupt rise of CellROX* fluorescence was observed for any of the cells. We also repeated the Amplex Red/APEX2 experiments in fermentation conditions using 4 μM LL-37. For all 117 cells studied, we observed no significant resorufin fluorescence signal, either within the cells or in the extracellular background (example in Fig 5A).

We also carried out experiments at 16 μM LL-37 in fermentation conditions, a concentration equal to the 6-hr MIC. Under those conditions, all cells shrink within 30 min. A weak signal from CellROX* again rose gradually over 30 min, but there was no abrupt increase and the average maximum signal after 30 min was comparable to that at 4 μM LL-37 in fermentation conditions (Fig 3A).

To summarize, in fermentation conditions, the CM (and presumably the OM) of a subset of cells is permeabilized at 4 μM LL-37 and the CM of all cells is permeabilized at 16 μM LL-37. There is no evidence of the same type of rapidly rising CellROX* and resorufin signals of oxidative stress that were observed in aerobic growth.

### Smaller signals of oxidative stress in growth under anaerobic respiration

Cells growing in EZRDM in the absence of oxygen but in the presence of glucose and added NO_3_^–^ carry out anaerobic respiration using the terminal reductase NarGHI, which reduces NO_3_^–^ to NO_2_^–^ [31]. To test whether a functional electron transport chain is sufficient to enable LL-37 to induce the CellROX* and resorufin signals, we carried out analogous fluorescence microscopy experiments on cells growing anaerobically in EZRDM supplemented with 10 mM KNO_3_. In anaerobic respiration conditions, 4 μM LL-37 induced CM permeabilization to Sytox Green in a significantly smaller fractions of cells than in aerobic growth conditions, on both the 30-min and 60-min timescales (Fig 1C). This is congruent with the increase in MIC (Table 2). On average, the maximum CellROX* signal generated over 30 min was fivefold smaller than in the peak signal in aerobic growth (Fig 3A). No resorufin signal, either intracellular or extracellular, was observed from the Amplex Red assay in any of 49 cells studied. At 16 μM LL-37 (higher than the MIC of 12 μM under anaerobic respiration conditions), all cells exhibited cytoplasmic membrane permeabilization within 15 min. We carried out the Amplex Red assay at this higher LL-37 concentration and again observed no signal whatsoever in any of the 45 cells studied.

### Magnitude of CellROX* signals for melittin, cecropin A, and indolicidin

For *E*. *coli* in aerobic conditions, on addition of 10 μM melittin (twice the aerobic MIC) we observed a strong, rapidly rising CellROX* fluorescence signal (S5 Fig). Much weaker, more slowly rising CellROX* fluorescence was observed (S5 Fig) on addition of 0.9 μM cecropin A (1X the aerobic MIC) and of 32 μM indolicidin (1X the aerobic MIC). The average maximum intensity results are shown in the bar graph of Fig 3B. These data are congruent with a strong increase in MIC from aerobic to anaerobic (fermentation) conditions for melittin, but not for cecropin A nor for indolicidin.

## Discussion

This work extends our earlier study of the attack of LL-37 on *E*. *coli* [19] to include direct observation of the timing of fluorescence signals that monitor oxidative stress. As shown before, the initial step in the attack is binding of the cationic LL-37 to the anionic lipopolysaccharide (LPS) layer, followed by permeabilization of the outer membrane (OM). For LL-37, we observed significant increases in MIC for cells growing under fermentation and anaerobic respiration conditions compared with aerobic respiration (Tables 1 and 2). In and of themselves, differences in MIC under different growth conditions should be interpreted cautiously. In addition to modulating oxidative stress effects, different growth conditions may also modulate the bacterial membrane composition. Such changes could alter the binding propensity of an AMP for the outer membrane and also the surface concentration of AMP required for membrane permeabilization.

The present work shows that for cells growing aerobically, an early green CellROX* signal gradually rises as the LL-37 concentration builds up in the periplasm, but before LL-37 has permeabilized the cytoplasmic membrane (CM) to the small dye Sytox Orange (Fig 4). Compared with the WT strain, the maximum CellROX* signal decreases almost 4-fold in the Δ*cydAB* deletion mutant but increases slightly (nominal 1.3-fold) in the Δ*cyoABCDE* deletion mutant. The signal is attenuated almost 6-fold after pre-treatment with KCN, which is known to inhibit aerobic respiration. The signal level was unchanged using the *D* stereoisomer form of LL-37. Entry of LL-37 into the periplasm gives the AMP access to the outer leaflet of the CM and to external surfaces of cytoplasmic membrane proteins.

**Fig 6:**
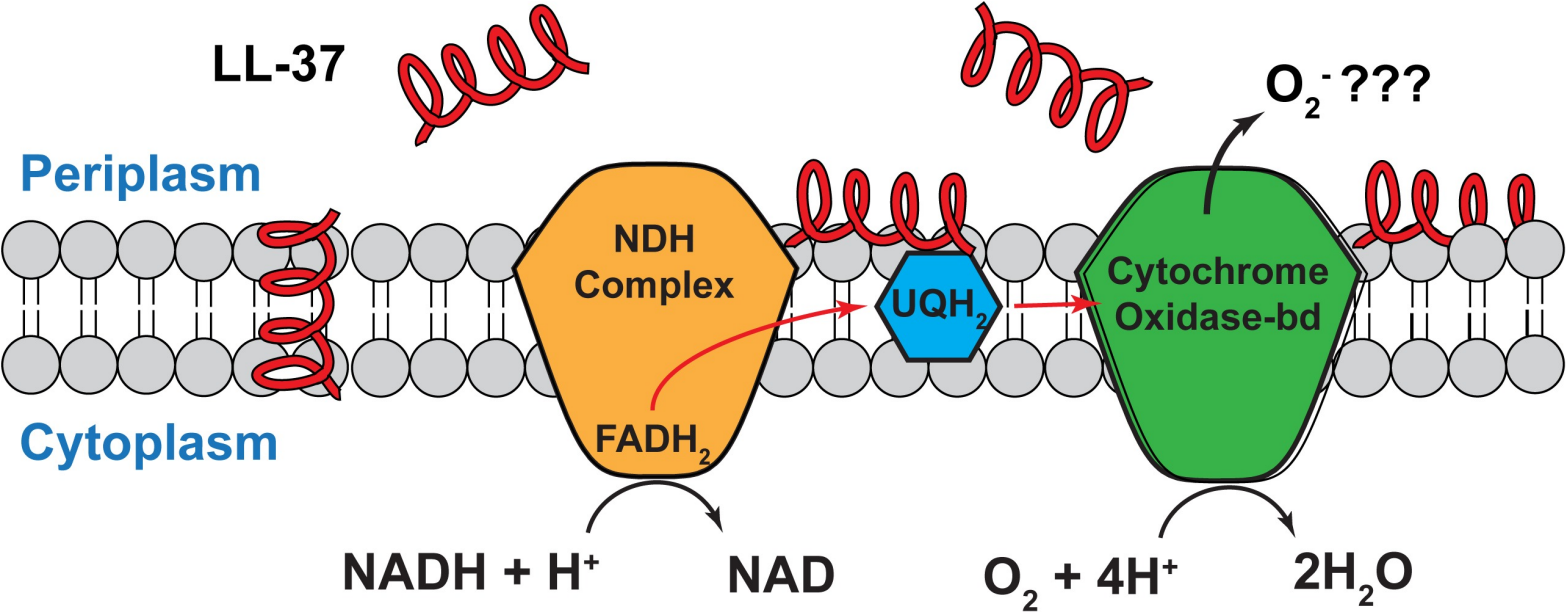
Schematic of the branch of the aerobic electron transport chain of *E*. *coli* terminating at cytochrome oxidase-*bd*. We suggest that LL-37 enters the periplasm, binds to the outer leaflet of the cytoplasmic membrane, and disrupts the proper activity of the terminal cytochrome oxidase-*bd* complex, releasing the intermediate superoxide (•O_2_^–^) into the periplasm.

These observations are consistent with a proposed mechanism in which LL-37 interferes with the terminal cytochrome oxidase-*bd*, causing inappropriate release of superoxide (•O_2_^–^) into the periplasmic space, where it oxidizes CellROX to CellROX* (Fig 6). Similarly, *in vitro* studies showed that the cyclic, cationic antimicrobial agent gramicidin S interfered with the activity of cytochrome oxidase-*bd*, but not with cytochrome oxidase-*bo*_3_ [34]. The interference might arise from direct interaction of LL-37 with the oxidase. Observation of the same CellROX* signal level using the *L-* or *D-* enantiomer of LL-37 argues against existence of a specific binding pocket within the cytochrome oxidase-*bd* structure; it does not rule out a non-specific interaction due to electrostatic binding, for example. Alternatively, LL-37 may disrupt -*bd* function indirectly by perturbation of the membrane environment, perhaps by strong interaction of the polycationic peptide with anionic lipids such as cardiolipin (CL) or phosphatidylglycerol (PG) [35].

Importantly, we know from inadvertent experiments that use of old CellROX samples induces cytoplasmic fluorescence in *E*. *coli* even without addition of LL-37. Presumably CellROX had already been oxidized to the fluorescent form CellROX*. This makes it plausible that CellROX* created by LL-37 action in the periplasm is able to permeate the intact cytoplasmic membrane, bind to DNA, and fluoresce, prior to permeabilization of the CM to Sytox Orange or presumably to LL-37 itself.

The subsequent CM permeabilization event enables Sytox Orange and presumably LL-37 itself to enter the cytoplasm. CM permeabilization correlates in time with abrupt, partial quenching of the CellROX* fluorescence (by an unknown mechanism, Fig 4A) and the abrupt onset of resorufin fluorescence (Fig 5B), presumed to be formed by reaction of Amplex Red with APEX2 and H_2_O_2_. The resorufin signal is detected primarily outside the cells, but some 20% of cells retain substantial resorufin inside the cytoplasm. The detailed mechanism of H_2_O_2_ production is unclear. The most plausible source is dismutation of •O_2_^–^ by the superoxide dismutases (SODs) that reside in the cytoplasm. Once LL-37 has permeabilized the CM to Sytox Orange and to LL-37 itself, the •O_2_^–^ formed in the periplasm may be able to cross the CM and reach the cytoplasm, where it finds SODs. Alternatively, if the CM becomes permeable to globular proteins, both the SODs and also APEX2 may pass from the cytoplasm to the periplasm, where they find •O_2_^–^ and produce H_2_O_2_ (which itself is permeable). In addition, once LL-37 enters the cytoplasm it may induce formation of additional •O_2_^–^ by some alternative mechanism.

Based on the properties of the dyes *in vitro*, we tentatively attribute the CellROX* signal that rises before CM permeabilization to production of superoxide (•O_2_^–^) and the resorufin signal that rises after CM permeabilization to production of hydrogen peroxide (H_2_O_2_). However, enhancement of intracellular oxidants other than •O_2_^–^ (or •OH) might cause conversion of CellROX Green to CellROX* [27]. The specificity of the APEX2 enzymatic reaction with H_2_O_2_ and Amplex Red to form resorufin lends support to the assumption that resorufin fluorescence is signaling an increase in hydrogen peroxide flux.

We found considerable evidence that aerobic respiration or a robust transmembrane potential or both are prerequisites for LL-37 to induce the early, gradually rising CellROX* signal and the delayed, abruptly rising resorufin signals (Figs 2–5). In Fig 3 we compare the maximum CellROX* intensity observed over 30 min for various conditions. Both oxidative stress signals are greatly attenuated by pre-treatment of cells with cyanide (which blocks O_2_ binding to the cytochrome oxidases, halts growth, and diminishes the pmf); by pre-treatment of cells with the protonophore CCCP (which abrogates the pmf); and by exclusion of oxygen from the medium, both in fermentation conditions (no operative electron transport chain) and in anaerobic respiration using NO_3_^–^ in the medium (enabling a different active electron transport chain).

It could be that the oxidants induced on entry of LL-37 to the periplasm enhance the ability of LL-37 to permeabilize the CM. Alternatively, the transmembrane electric field associated with the strong aerobic pmf points in the direction that would enhance the ability of a cationic peptide such as LL-37 to penetrate the low dielectric core of the bilayer and its substantial, repulsive dipole potential [36]. Such an effect was observed previously for cationic antibiotics such as gentamicin [37]. In anaerobic fermentation (Figs 1C, 2C, 2D and 5A) and anaerobic respiration conditions (Fig 1C), the pmf is likely lower than in aerobic growth [24]. In those lower-pmf conditions, 4 μM LL-37 permeabilizes the CM and halts growth in a smaller fraction of cells. Even those permeabilized cells do not exhibit the type of CellROX* or resorufin signals characteristic of the rapid oxidative stress induced in aerobic conditions.

It is difficult to pinpoint exactly what aspects of the attack of LL-37 on *E*. *coli* cause the halting of growth and the eventual killing of cells. The smaller MIC in aerobic vs anaerobic growth conditions (Table 1) suggests that the observed oxidative stress events contribute, perhaps indirectly. Permeabilization of the CM to small species and concomitant loss of the pmf is undoubtedly an important factor as well. In a recent study of *E*. *coli* attacked by an analogue of the AMP PMAP-23, at killing concentrations the copy number of AMP per cell was estimated to be 10^6^–10^7^, and the assay was most sensitive to membrane-bound copies [38]. For the synthetic cationic peptide “ARVA”, the analogous copy number was estimated to be >10^8^ per cell [39]. Even at 10^6^ per cell, the AMP concentration would be 1 mM. If LL-37 also binds to *E*. *coli* at such high concentrations, it is easy to imagine multiple harmful processes occurring in parallel.

The generality of oxidative stress induction by AMP action on bacteria deserves further exploration. As judged by the MIC in aerobic growth vs fermentation conditions (Table 1), the efficacy against *E*. *coli* of LL-37 and melittin depends on oxygen levels, while that of cecropin A and indolicidin does not. In previous work, we observed oxygen-sensitive MICs and time-dependent intra-cellular fluorescence signals indicative of oxidative stress during the attack on MG1655 *E*. *coli* by the synthetic AMP CM15 [20] and by the synthetic, highly cationic random β-peptide copolymer **MM**_**63**_:**CHx**_**37**_ [22]. In the present work, the oxidative signals induced by LL-37 decreased significantly on deletion of cytochrome oxidase-*bd*, but not on deletion of the–*bo*_3_ oxidase. This suggests a remarkably specific target of LL-37 activity. It seems likely that different AMPs will prove to induce oxidative stress by different mechanisms.

Finally, the enhancement of growth-halting effects of LL-37 in aerobic vs anaerobic conditions suggests the possibility that the degree of oxygenation in specific tissues may help to regulate AMP activity. For example, in the human gut the fraction of strict anaerobes increases from proximal to distal; in the colon, the oxygen partial pressure is only 25% of that in the atmosphere [23]. An earlier study of human β–defensin-1 found the AMP to be much more potent in its reduced, unfolded form against the pathogenic fungus *Candida albicans* and against anaerobic, Gram positive commensals of the *Bifidobacterium* and *Lactobacillus* species [23]. The effect was specific to certain microbial species. In contrast, human β-defensin-3, which is extremely potent in its oxidized, folded form, was less potent under reducing conditions. Like LL-37, hBD-3 is more potent in aerobic conditions. More work is needed, but it is already evident that the degree of oxygenation affects different human AMPs in different ways. Future work will test the generality of the induction of oxidative stress by other natural AMPs.

## Materials and methods

### Bacterial strains, materials, and growth conditions

The strains are listed in Table 2. The background (“WT”) strain is MG1655 (K12) in all cases. Experiments on periplasmic GFP used strain JCW10, in which TorA-GFP is expressed from plasmid pJW1 as previously described [26]. TorA-GFP is transported to the periplasm by the twin-arginine transport system and the TorA signal peptide is cleaved, leaving free GFP in the periplasm. ZY01 is the strain that expresses the peroxidase APEX2 from a plasmid introduced into the background strain, as described previously [20]. To construct the deletion mutant strain called Δ*cyoABCDE*, lacking the gene for cytochrome-*bo*_3_ oxidase, we performed λ-Red reconstruction, replacing the *cyoABCDE* gene with a kanamycin resistance gene. The deletion of the *cyoABCDE* gene and the replacement by a kanamycin resistance gene were confirmed by PCR and DNA sequencing. The deletion mutant strain Δ*cydAB* was constructed and confirmed analogously. To visualize resorufin generation in the Δ*cyoABCDE* deletion mutant, we transformed a pASK-IBA3plus vector containing the APEX2 gene into *ΔcyoABCDE*, yielding strain ZY02.

Unlabeled LL-37 lacking a C-terminal amide was purchased from Anaspec (61302). Rhodamine B-LL-37 (no C-terminal amide) was purchased from Bachem (4049885). The oxidation sensitive dye CellROX Green (C10444) and Amplex Red (A22188) were purchased from Invitrogen. The DNA stains Sytox Green (S7020) and Sytox Orange (S11368) were purchased from Thermo-Fisher Scientific.

Bulk cultures were grown in EZ rich, defined medium (EZRDM) [40], which is a MOPS-buffered solution at pH = 7.4 supplemented with metal ions (M2130; Teknova), glucose (2 mg/mL), amino acids and vitamins (M2104; Teknova), nitrogenous bases (M2103; Teknova), 1.32 mM K_2_HPO_4_, and 76 mM NaCl. Cultures were grown from glycerol frozen stock to stationary phase overnight at 30°C. Subcultures were grown to exponential phase (OD = 0.2–0.6 at 600 nm) at 30°C before sampling for the microscopy experiments.

### Minimum inhibitory concentration (MIC) assay

The aerobic MIC values for the various AMPs (Table 1) were determined using the broth microdilution method as previously described [19]. Two-fold serial dilutions of LL-37 in 1× EZRDM were performed in separate rows of a polystyrene 96-well plate, with each plate containing an inoculum of *E*. *coli* MG1655. The inoculum was a 1:20 dilution from a bulk culture at midlog phase (OD600 = 0.5) grown at 30°C. The plate was incubated at 30°C and shaken at 200 rpm in a Lab-Line Orbital Environ Shaker (model 3527) for 6 hr. The MIC value was taken as the lowest concentration for which no growth was discernible (<0.05 OD) after 6 hr.

Anaerobic MIC values (Table 1) were measured on a 96-well plate that was sealed with plastic wrap. Cells were incubated in EZRDM containing protocatechuic acid (PCA) at 10 mM and protocatechuate 3,4-dioxygenase (PCD) at 100 nM to scavenge oxygen [41]. The plate was incubated at 30°C for 6 hr, followed by OD measurements. In the earlier study of CM15 [20], we tested that PCA by itself does not interfere with CellROX* fluorescence.

### Time-lapse recovery assay

The time-lapse recovery assays utilized an MG1655 culture in bulk. Overnight culture of wild-type MG1655 was inoculated at 1:100 dilution in 2 mL EZRDM at 30°C. When the culture is at midlog phase (OD600 = 0.5), the culture was diluted with warmed EZRDM to 1:10 and incubated with different concentrations (0, 4, 8, and 16 μM) of LL-37. 100 uL of each culture was sampled at different time point (30 min, 1-hr. and 2-hr incubation). Then, each culture was 10-fold serial diluted with warmed EZRDM into a 96-well plate. Each dilution was plated into fresh LB agar plates and the plates were incubated at 30°C for 24 hr. The plates were then visually inspected for growth of colonies. The control procedure was the same except that the LL-37 was omitted. See S1 Fig for results.

### Microfluidics chamber for aerobic and anaerobic microscopy

As previously described [20], imaging of individual cells was carried out at 30°C in a microfluidics chamber consisting of a single rectilinear channel of uniform height of 50 μm and width of 6 mm, with a channel length of 11 mm. The total chamber volume is ~10 μL. After bonding of the PDMS chamber to the glass coverslip, 0.01% poly-L-lysine (molecular weight >150,000 Da) was injected through the chamber for 30 min and rinsed thoroughly with Millipore water. *E*. *coli* cells are immobilized on the coverslip but grow normally. During imaging experiments, the chamber was maintained at 30°C with an automatic temperature controller.

For aerobic imaging experiments, the medium is exposed to air over three hr while held at 30°C in a shaker bath; this ensures full oxygenation of the medium. In addition, the PDMS ceiling of the microfluidics device is permeable to the ambient gases N_2_ and O_2_. For anaerobic imaging experiments, O_2_ must be prevented from entering the chamber through its ceiling. A small anaerobic chamber surrounding the microfluidics device was constructed of aluminum with a nitrogen gas inlet and outlet. Details are provided elsewhere [20]. Before injection of cells, nitrogen gas flowed through the chamber continuously for 1.5 hr. *E*. *coli* were grown in aerobic conditions until injected into the chamber. Fresh deoxygenated EZRDM was made by treating EZRDM with 50 nM protocatechuate 3,4-dioxygenase (PCD) and 2.5 mM protocatechuic acid (PCA). This was used to wash the cells at 30°C before plating. Deoxygenated EZRDM (with or without addition of 10 mM KNO_3_) then flowed across the plated cells for 30 min before injection of antimicrobial peptides and CellROX. The subsequent microscopy imaging experiment was carried out as before.

### Microscopy

Single-cell imaging was performed on two different microscopes: a Nikon TE300 inverted microscope with a 100×, 1.3 N.A. phase contrast objective and a Nikon Eclipse Ti inverted microscope with a 100×, 1.45 N.A. phase contrast objective. For the TE300, images were further magnified 1.45× in a home-built magnification box. GFP, Sytox Green, and CellROX* were imaged using 488 nm excitation (Coherent Sapphire laser), expanded to illuminate the field of view uniformly. The emission filter was HQ525/50 (Chroma Technology). Resorufin and Sytox Orange were imaged using 561 nm excitation (Coherent Sapphire laser). The emission filter was HQ617/73 (Chroma Technology). Laser intensities at the sample were typically ~5 W/cm^2^ at 488 nm and ~2.5 W/cm^2^ at 561 nm. Fluorescence images were obtained with an EMCCD camera, either Andor iXon 897 or Andor iXon 887. In both cases, the pixel size corresponds to 110 ± 10 nm at the sample.

For single color experiments, time-lapse movies of 60-min total duration were obtained as 600 frames of 50-ms exposure time each, with fluorescence and phase contrast images interleaved at 6-s intervals (12 s per complete cycle). For dual color experiments, μManager was used to obtain the data and switch filters between frames using a LB10-NW filter wheel (Sutter). The time-lapse movies of 35-min total duration were obtained as 1050 frames of 50-ms exposure time each, with green fluorescence (488 nm excitation), red fluorescence (561 nm excitation), and phase contrast images interleaved (6 s per complete cycle). To minimize spectral bleed-through in the two-color experiments, we utilized the narrower filters HQ510/20 for the green channel and HQ600/50M for the red channel.

### CellROX Green oxidation assay

CellROX Green (Life Technologies) is a proprietary oxidation-sensitive dye whose fluorescence quantum yield at 500–550 nm after excitation at 488 nm increases dramatically on oxidation in the presence of ds-DNA. It readily permeates both *E*. *coli* membranes. The manufacturer tested its sensitivity to different reactive oxygen species in the presence of ds-DNA *in vitro* including hydroxyl radical (•OH), superoxide (O_2_^–^), hydrogen peroxide (H_2_O_2_), peroxynitrite (ONOO^–^), nitric oxide (NO), and hypochlorite (ClO^–^). The only two oxidizing agents that significantly enhanced CellROX* fluorescence were hydroxyl radical and superoxide. Importantly, hydrogen peroxide has no effect.

In the CellROX* imaging experiments, MG1655 cells were injected into the microfluidics chamber. After allowing 5 min for plating of cells, the bulk solution was washed away with fresh, pre-warmed, aerated EZRDM. After the wash, cells were grown for 5 min prior to the injection of 4 μM LL-37 plus 2.5 μM CellROX Green. To maintain good aeration and steady bulk concentrations, the medium with LL-37 and CellROX Green flowed continuously at 0.3 mL/hr.

### Amplex Red oxidation assay

As previously described [20], the assay for single-cell, time-resolved measurement of H_2_O_2_ production following LL-37 treatment is based on the well-established Amplex Red method [30]. Some peroxidases (but not the catalases naturally occurring in *E*. *coli*) catalyze reaction of the dye Amplex Red with H_2_O_2_ to form the red fluorescent species resorufin (λ_em_ = 585 nm). Recently Collins and coworkers [29] adapted the method to carry out the Amplex Red + H_2_O_2_ reaction inside the cytoplasm by inserting a plasmid that expresses the peroxidase APEX2 (mutated ascorbate peroxidase). Their method detects H_2_O_2_ produced inside the cell using plate-based bulk fluorescence measurements with time resolution of ~60 min. Here we use intracellular APEX2 combined with single-cell, time-resolved detection by fluorescence microscopy. This enables sensitive detection of intracellular H_2_O_2_ production with 12-s time resolution and correlation of LL-37-induced H_2_O_2_ production with other events in real time.

## Supporting information

S1 Fig Bactericidal assay. Cultures growing aerobically in EZRDM at 30°C were pre-treated with 1 mM KCN for 5 min (+KCN) or not (–KCN) and incubated with LL-37 at 0 or 4 μM (1X the aerobic MIC) for 30 min (left) or 1 hr (right). The cultures were then sampled and diluted before spotting onto LB plates for overnight incubation at 30°C. From left to right in each panel, serial dilutions led to addition of 5 x 10^6^, 5 x 10^5^, 5 x 10^4^, 5 x 10^3^, 5 x 10^2^, and 5 cells/mL onto the LB plate. Colonies were photographed the next morning. There is a higher cell survival rate for 30-min LL-37 treatment than for 60-min and after KCN pre-treatment than without KCN pre-treatment.
(TIF)

S2 Fig Effects of 4 μM LL-37 (the 6-hr aerobic MIC) on MG1655 *E*. *coli* pretreated with 1 mM KCN for 5 min. a) Example of CellROX* and Sytox Orange fluorescence intensity vs time after the injection of LL-37. b) Comparison of mean, single-cell CellROX* peak fluorescence intensity without and with KCN pre-treatment. Error bars are ±1 SD of the mean. c) Cumulative distribution function for cells that undergo cytoplasmic membrane permeabilization (CMP) after the injection of 4 μM LL-37 without (90 cells) and with (60 cells) KCN pre-treatment, as judged by Sytox Orange fluorescence.
(TIF)

S3 Fig Time dependence of background fluorescence (outside of any cells) from resorufin after injection at *t* = 0 of 4 μM LL-37 plus 10 μM Amplex Red across cells expressing APEX2 and growing aerobically. (TIF)

S4 Fig (A) CellROX* total fluorescence intensity vs time within a single, representative Δ*cyo*-*ABCDE* cell. Flow of 4 μM LL-37 and 2.5 μM CellROX Green begins at *t* = 0. (B) CellROX* total fluorescence intensity vs time within a single, representative Δ*cyd*-*AB* cell. Flow of 4 μM LL-37 and 2.5 μM CellROX Green begins at *t* = 0. Both signals are taken under identical laser and imaging conditions, so that quantitative comparison of signal amplitudes is appropriate. See Fig 3B for comparison of the mean of the peak CellROX* signal for WT, Δ*cyo*-*ABCDE*, and Δ*cyd*-*AB* strains.
(TIF)

S5 Fig CellROX* total fluorescence intensity vs time within single, representative WT cells for three additional AMPs. Flow of each AMP and 2.5 μM CellROX Green begins at *t* = 0. All three signals are taken under identical laser and imaging conditions, so that quantitative comparisons of signal amplitudes are appropriate. (A) 10 μM melittin (twice the aerobic MIC). (B) 0.9 μM cecropin A (1X the aerobic MIC). (C) 32 μM indolicidin (1X the aerobic MIC). See Fig 3B for comparison of the mean of the peak CellROX* signals for LL-37, melittin, cecropin A, and indolicidin.
(TIF)

S6 Fig Histogram of time lags between the peak of the CellROX* signal and the abrupt onset of the Sytox Orange signal. Addition of 4 μM LL-37, 2.5 μM CellROX Green, and 5 nM Sytox Orange were injected across plated cells growing aerobically. The time between the peak CellROX* signal and the onset of Sytox Orange fluorescence was measured, as shown in Fig 4A. Most often, the two events coincided in time within 1 min.
(TIF)

S7 Fig Example of single-cell resorufin fluorescence vs time in aerobic growth using the Δ*cyoABCDE* mutant strain including Amplex Red expressed from a plasmid. Injection of 4 μM LL-37 begins at *t* = 0.
(TIF)

S1 Movie *Left*: phase contrast images of a single *E*. *coli* cell expressing periplasmic GFP and growing in aerobic conditions. *Right*: green channel fluorescence, showing periplasmic GFP at early times (halo images) and Sytox Green at later times. Flow of 4 μM LL-37 and 5 nM Sytox Green was initiated at *t* = 0. Times in minutes as shown. Snapshots of 50 ms duration; 12 s per complete imaging cycle.
(AVI)

S2 Movie *Left*: phase contrast images of a single, wild-type *E*. *coli* cell growing in aerobic conditions. *Right*: green fluorescence images from CellROX*. Flow of 4 μM LL-37 and 2.5 μM CellROX Green was initiated at *t* = 0. Times in minutes as shown. Snapshots of 50 ms duration; 12 s per complete imaging cycle.
(AVI)

S3 Movie Two-color imaging of CellROX* (left) and Sytox Orange (right) for a single, wild-type *E*. *coli* cells growing in aerobic conditions. Flow of 4 μM LL-37, 2.5 μΜ CellROX Green, and 5 nM Sytox Orange was initiated at *t* = 0. Times in minutes as shown. Snapshots of 50 ms duration; 6 s per complete imaging cycle.
(AVI)

S4 Movie *Left*: phase contrast images of a single *E*. *coli* cell expressing APEX2 from a plasmid and growing in aerobic conditions. *Right*: red channel fluorescence from resorufin. Note the gradually increasing extracellular background signal. Flow of 4 μM LL-37 and 10 μM Amplex Red was initiated at *t* = 0. Times in minutes as shown. Snapshots of 50 ms duration; 12 s per complete imaging cycle.
(AVI)

S5 Movie Two-color imaging of Sytox Green fluorescence (left) and resorufin fluorescence (right) for a single *E*. *coli* cell expressing APEX2 from a plasmid and growing in aerobic conditions. Flow of 4 μM LL-37, 5 nM Sytox Green, and 10 μM Amplex Red was initiated at *t* = 0. Times in minutes as shown. Snapshots of 50 ms duration; 6 s per complete imaging cycle.
(AVI)
