# Supplementary figures and images for: Oxidative stress induced in E. coli by the human antimicrobial peptide LL-37
Source: PLoS Pathog. 2017 Jun 30;13(6):e1006481. doi: 10.1371/journal.ppat.1006481 (PMC5509375; doi:10.1371/journal.ppat.1006481)

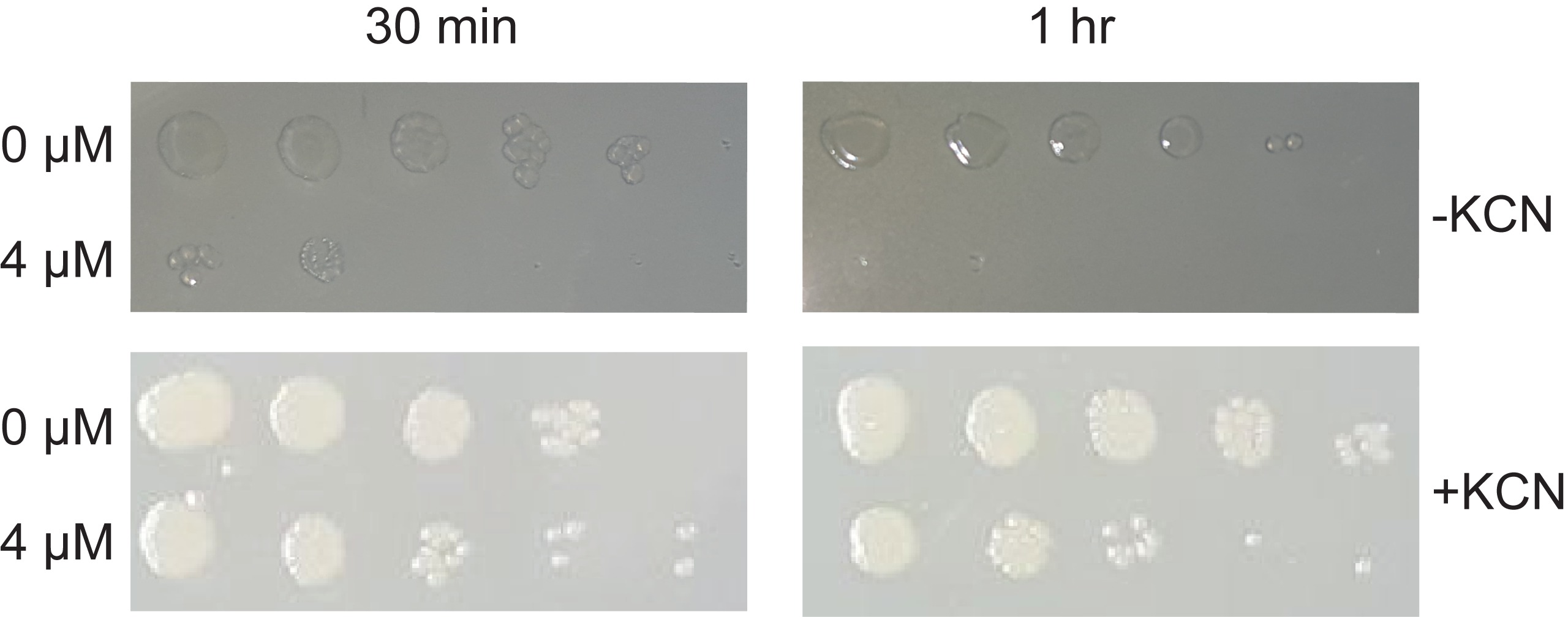

Supplement: S1 Fig — Cultures growing aerobically in EZRDM at 30°C were pre-treated with 1 mM KCN for 5 min (+KCN) or not (–KCN) and incubated with LL-37 at 0 or 4 μM (1X the aerobic MIC) for 30 min (left) or 1 hr (right). The cultures were then sampled and diluted before spotting onto LB plates for overnight incubation at 30°C. From left to right in each panel, serial dilutions led to addition of 5 x 10^6^, 5 x 10^5^, 5 x 10^4^, 5 x 10^3^, 5 x 10^2^, and 5 cells/mL onto the LB plate. Colonies were photographed the next morning. There is a higher cell survival rate for 30-min LL-37 treatment than for 60-min and after KCN pre-treatment than without KCN pre-treatment. (TIF) [file ppat.1006481.s001.tif]

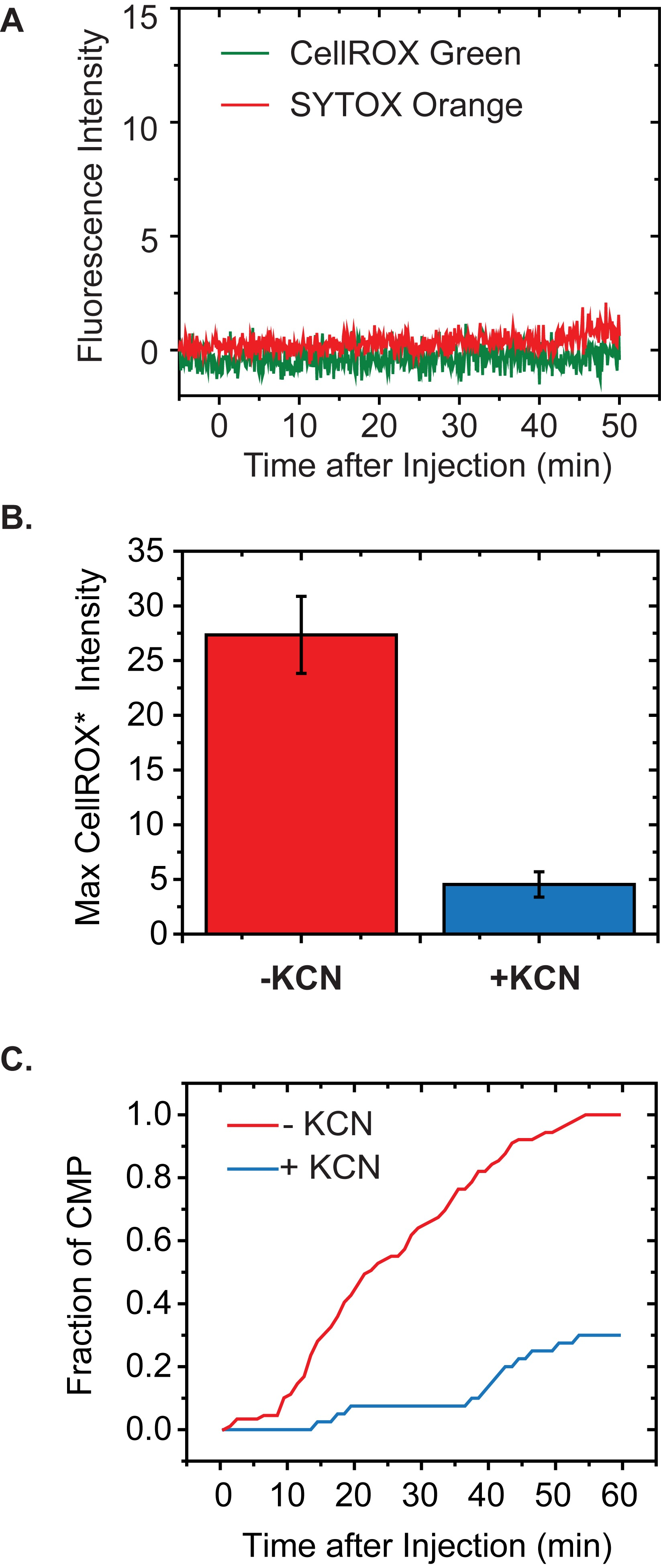

Supplement: S2 Fig — a) Example of CellROX* and Sytox Orange fluorescence intensity vs time after the injection of LL-37. b) Comparison of mean, single-cell CellROX* peak fluorescence intensity without and with KCN pre-treatment. Error bars are ±1 SD of the mean. c) Cumulative distribution function for cells that undergo cytoplasmic membrane permeabilization (CMP) after the injection of 4 μM LL-37 without (90 cells) and with (60 cells) KCN pre-treatment, as judged by Sytox Orange fluorescence. (TIF) [file ppat.1006481.s002.tif]

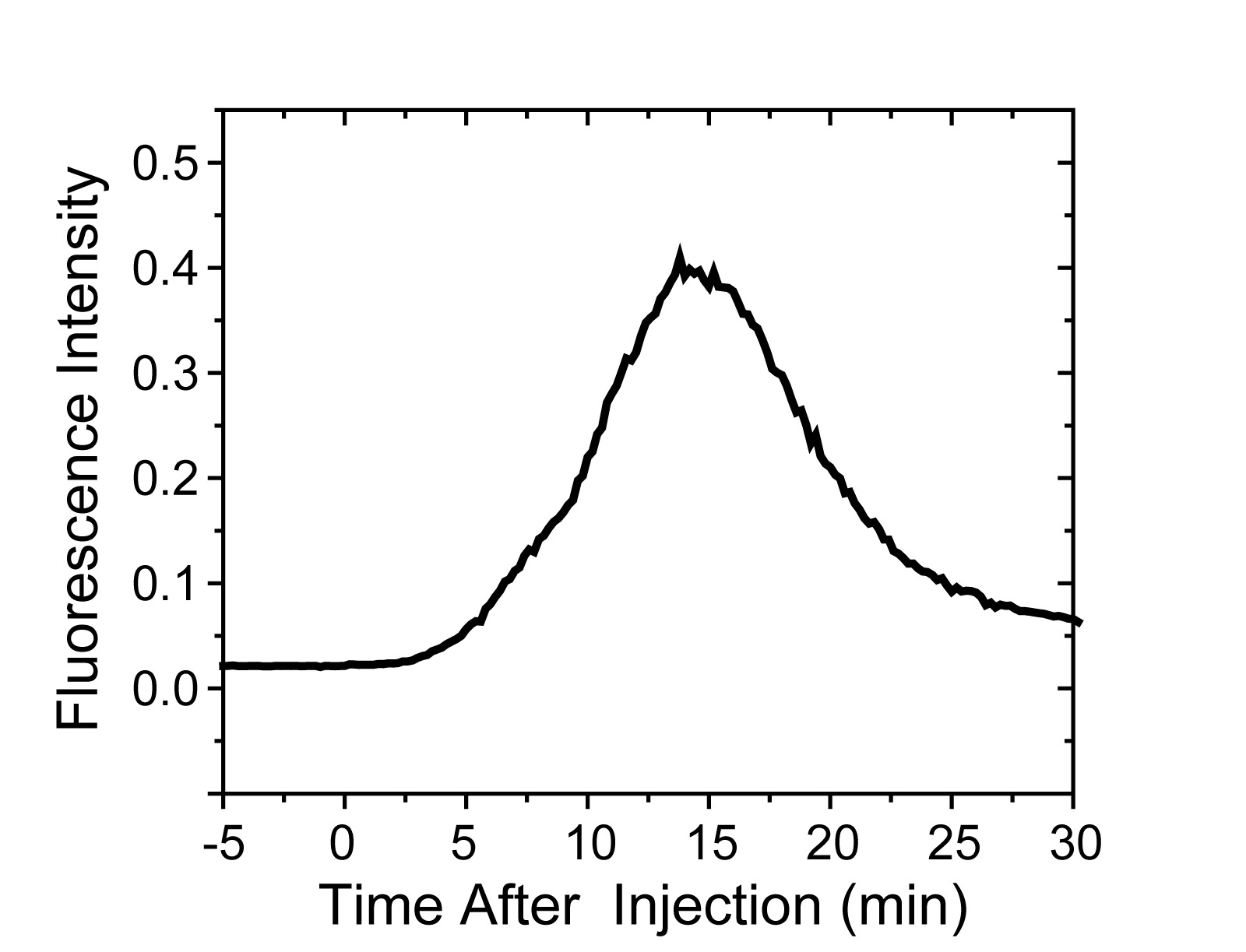

Supplement: S3 Fig — (TIF) [file ppat.1006481.s003.tif]

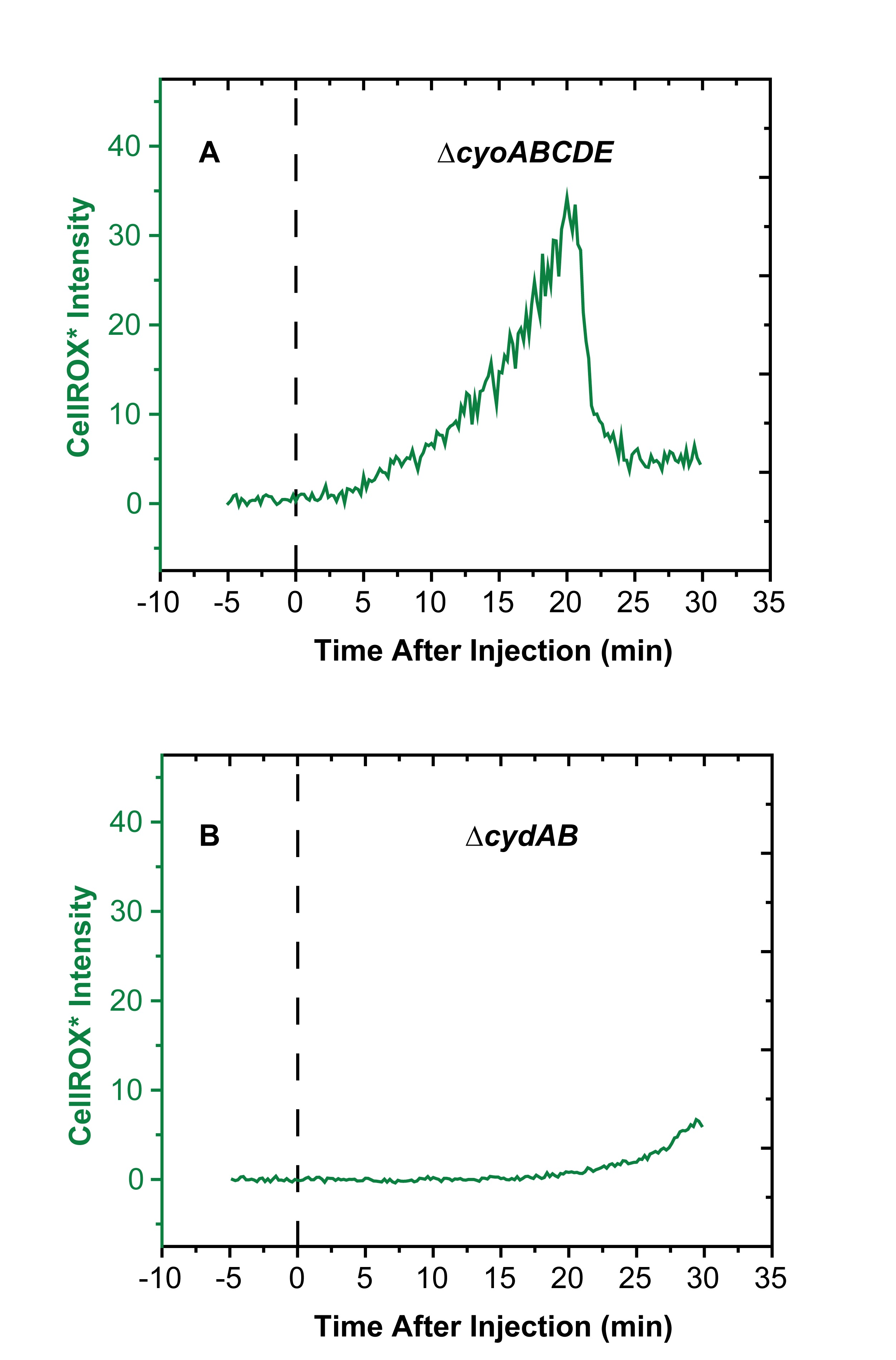

Supplement: S4 Fig — (A) CellROX* total fluorescence intensity vs time within a single, representative Δcyo-ABCDE cell. Flow of 4 μM LL-37 and 2.5 μM CellROX Green begins at t = 0. (B) CellROX* total fluorescence intensity vs time within a single, representative Δcyd-AB cell. Flow of 4 μM LL-37 and 2.5 μM CellROX Green begins at t = 0. Both signals are taken under identical laser and imaging conditions, so that quantitative comparison of signal amplitudes is appropriate. See Fig 3B for comparison of the mean of the peak CellROX* signal for WT, Δcyo-ABCDE, and Δcyd-AB strains. (TIF) [file ppat.1006481.s004.tif]

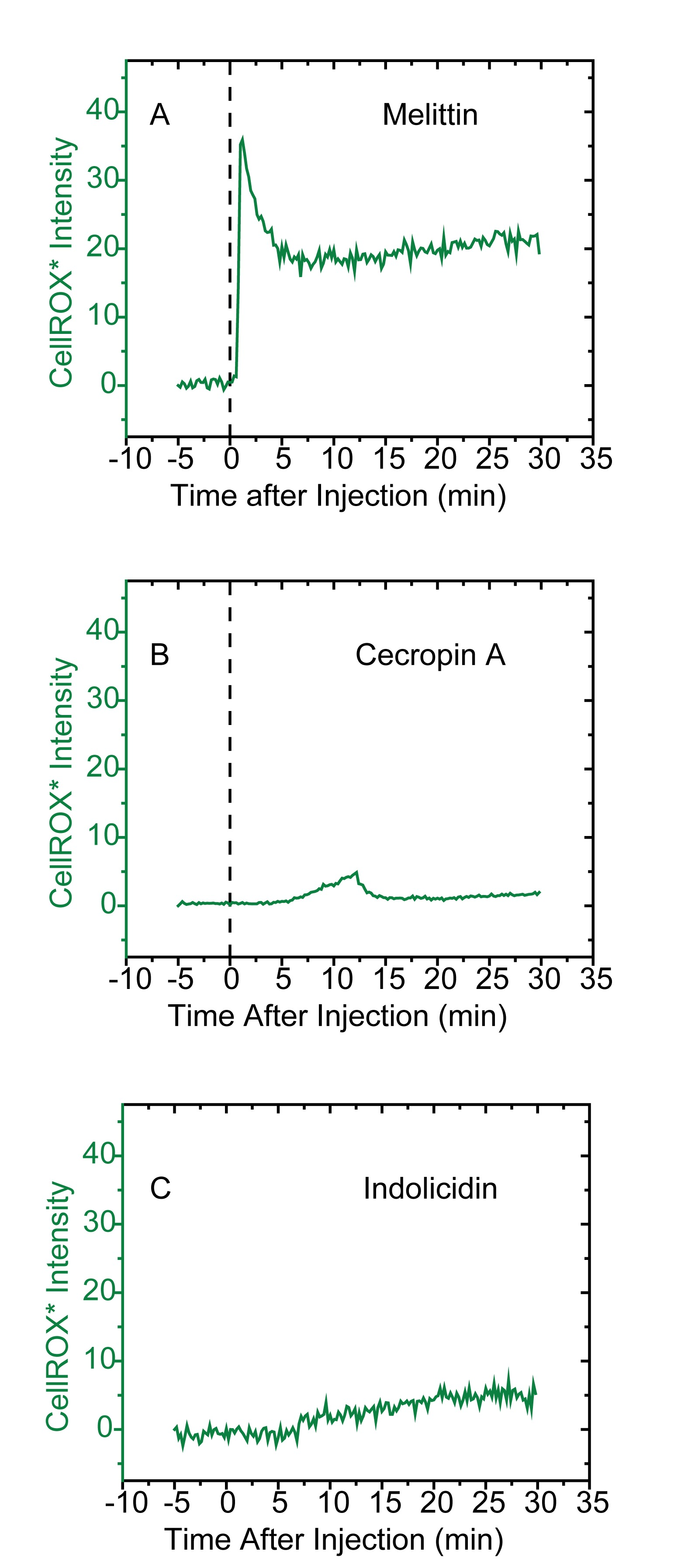

Supplement: S5 Fig — Flow of each AMP and 2.5 μM CellROX Green begins at t = 0. All three signals are taken under identical laser and imaging conditions, so that quantitative comparisons of signal amplitudes are appropriate. (A) 10 μM melittin (twice the aerobic MIC). (B) 0.9 μM cecropin A (1X the aerobic MIC). (C) 32 μM indolicidin (1X the aerobic MIC). See Fig 3B for comparison of the mean of the peak CellROX* signals for LL-37, melittin, cecropin A, and indolicidin. (TIF) [file ppat.1006481.s005.tif]

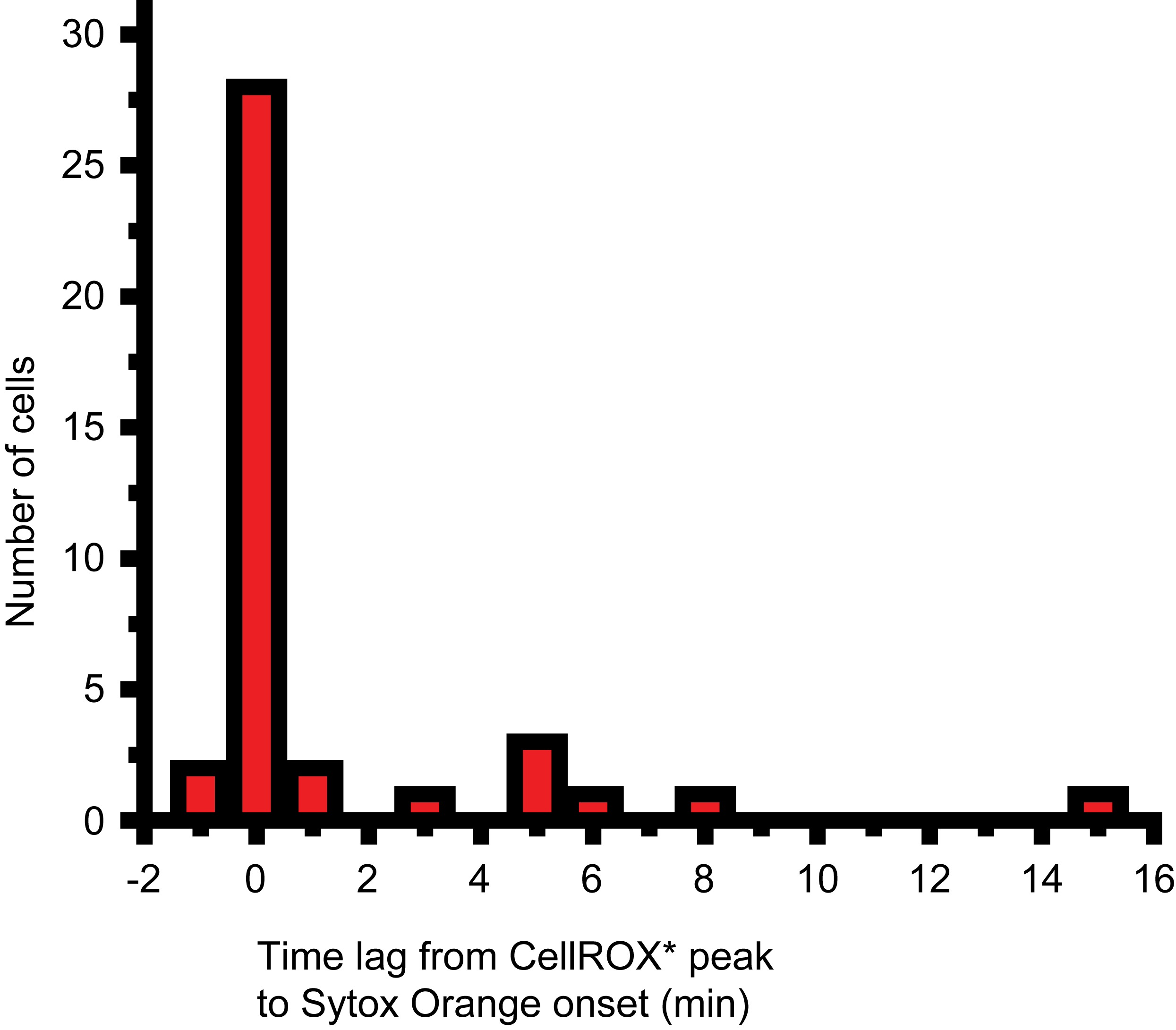

Supplement: S6 Fig — Addition of 4 μM LL-37, 2.5 μM CellROX Green, and 5 nM Sytox Orange were injected across plated cells growing aerobically. The time between the peak CellROX* signal and the onset of Sytox Orange fluorescence was measured, as shown in Fig 4A. Most often, the two events coincided in time within 1 min. (TIF) [file ppat.1006481.s006.tif]

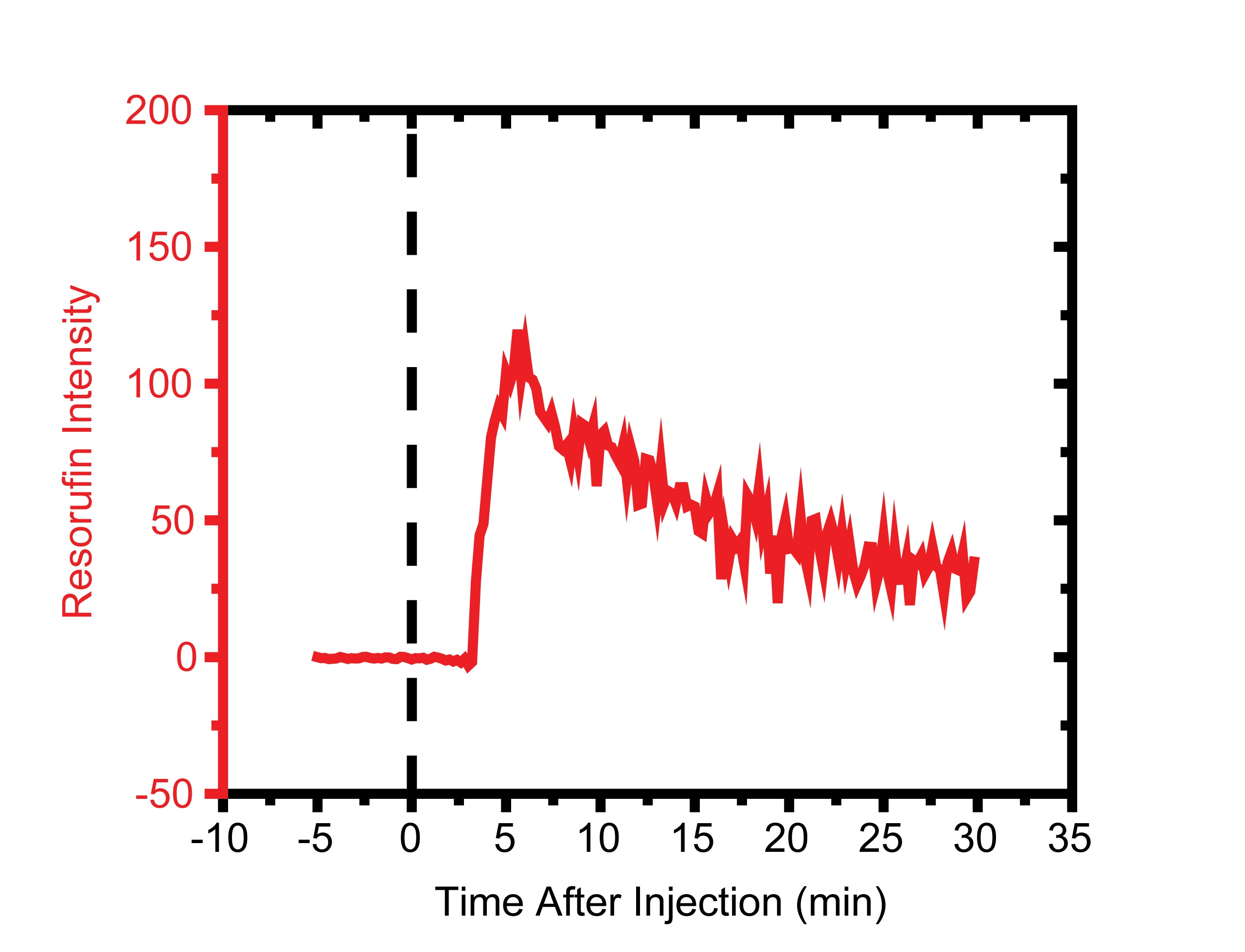

Supplement: S7 Fig — Injection of 4 μM LL-37 begins at t = 0. (TIF) [file ppat.1006481.s007.tif]
